# Transcription Profiling of Potato Leaves in Response to Heat Stress at Single‐Cell Resolution

**DOI:** 10.1111/pbi.70546

**Published:** 2026-01-13

**Authors:** Shiqi Wen, Ke Wang, Wenqian Liang, Rongrong Liu, Zihan Li, Xinlong Chen, Yan Li, Dianqiu Lv, Hongju Jian

**Affiliations:** ^1^ Integrative Science Center of Germplasm Creation in Western China (CHONGQING) Science City, Chongqing Technology Innovation Center of Breeding Southwest University Chongqing People's Republic of China; ^2^ College of Agronomy and Biotechnology Southwest University Chongqing People's Republic of China; ^3^ Chongqing Key Laboratory of Biology and Genetic Breeding for Tuber and Root Crops Southwest University Chongqing People's Republic of China; ^4^ Key Laboratory of Germplasm Innovation of Upper Yangtze River, Ministry of Agriculture and Rural Affairs Southwest University Chongqing People's Republic of China

**Keywords:** co‐expression network, heat stress, potato, snRNA‐seq, *StPIF4*

## Abstract

Potato ( *Solanum tuberosum* L.) is a globally important food crop with considerable nutritional and economic value. Heat stress significantly inhibits potato plant growth and tuber development, constraining the sustainable development of the potato industry. Currently, studies on the cellular‐level mechanisms underlying heat adaptation in potato remain relatively scarce. In this study, single‐nucleus RNA sequencing was employed to construct single‐cell transcriptomic maps of potato leaves under normal and heat stress conditions, yielding 77 344 high‐quality nuclei and identifying six major cell types. The results indicated that epidermal cells represented the key cell type in heat‐stress response, exhibiting the highest number of differentially expressed genes, whereas vascular cells were positioned in the transition zone of the pseudo‐time trajectory and may have been involved in cell differentiation processes. By integrating bulk RNA‐seq data, a heat stress response co‐expression network was constructed, identifying 12 core transcription factors, with *StPIF4* appearing repeatedly. Experimental validation confirmed that heat stress strongly induced *StPIF4* expression. Functional studies demonstrated that *StPIF4* significantly enhanced potato heat tolerance by improving reactive oxygen species scavenging capacity. This study provided cellular‐level insights into the mechanisms underlying potato adaptation to heat stress.

## 1 Introduction

Potato ( *Solanum tuberosum* L.), a globally important food crop, plays a crucial role in global food security and economic development (Gao et al. 2019; Jiang et al. 2022). However, with the intensification of global warming, the frequency of extreme heat events continued to increase, making heat stress a primary environmental factor limiting crop productivity and quality improvement. Heat stress has been shown to inhibit plant photosynthesis, reduce carbohydrate synthesis, and increase transpiration, leading to water imbalance, leaf chlorosis, and plant mortality. This significantly shortened the life cycle of crops and reduced yield (Li, Jiang, et al. 2024; Yang et al. 2020). For potato, temperature is among the most difficult external factors to regulate during growth and development. Heat stress not only inhibited plant growth and tuber enlargement but also directly reduced tuber yield and quality (Herman et al. 2017; Tang et al. 2018, 2016; Yan et al. 2016; Zhao et al. 2017). Consequently, identifying key heat‐tolerant genes in potato, elucidating the mechanisms by which heat stress influences potato physiology, and revealing the response mechanisms to heat stress are of critical importance for potato breeding.

The mechanisms underlying responses to heat stress have become a major research focus in plant science. Among these, epidermal cells (ECs), as primary sensors of external temperature, play a critical role in heat‐stress response. They regulate stomatal movement to balance body temperature and water loss while also responding to heat shock signalling pathways, thereby inducing the expression of heat shock proteins (HSP) and antioxidants (Kan et al. 2023; Xu et al. 2025). Furthermore, ECs modulate the synthesis of the cuticle and epidermal waxes, thus enhancing the plant's tolerance to elevated temperatures (Lu et al. 2025). Elevated temperatures induce the conversion of phytochrome B (phyB) from its active form Pfr to its inactive form Pr. This shift releases the inhibition exerted by Pfr dimers on the thermomorphogenesis regulators PIF4 and PIF7, thereby activating downstream signalling pathways (Koini et al. 2009; Kumar et al. 2012). Heat‐shock factor (HSF) and HSP genes play indispensable roles in the transcriptional regulation of heat responses. For instance, the HsfA1 protein, a member of the Arabidopsis HSF family, functions as a primary activator of heat‐stress‐responsive genes and substantially enhances plant heat tolerance (Ding et al. 2020; Ohama et al. 2017). In potato, overexpression of *StHsfA2* not only enhances plant heat tolerance but also partially alleviates the suppression of tuber yield by heat stress, thereby contributing to the recovery of yield and starch content (Zhang et al. 2025). Overexpression of *StERF94* further enhances potato adaptation to heat stress (Charfeddine et al. 2023). But, the role of *StPIF4* in the response to heat stress remains uncharacterized in potato.

Heat stress induced the accumulation of reactive oxygen species (ROS) within plants. When excess ROS accumulated and caused cellular damage, antioxidant and ROS‐scavenging systems were triggered to maintain cellular redox homeostasis and to support adaptation to thermal stress (Choudhury et al. 2017). Extracellular H_2_O_2_ acted as a signalling molecule, directly activating associated sensors. For example, leucine‐rich repeat receptor kinase HPCA1 sensed H_2_O_2_ via its cysteine residues, thereby promoting an increase in Ca^2+^ levels (Wu et al. 2020). Research indicated that *StGATA2* enhanced potato heat stress tolerance by increasing antioxidant enzyme activities—superoxide dismutase (SOD), catalase (CAT), and peroxidase (POD)—and by reducing hydrogen peroxide content (Zhu et al. 2023). However, it remained unclear whether these sensors were involved in the heat‐stress response.

In plants, cellular functional specialisation arose through the differential regulation of shared genomic information, resulting in cell type‐specific transcriptional signatures. Single‐cell RNA sequencing (scRNA‐seq) served as an effective tool for investigating this field. Since the initial application using native protoplasts in Arabidopsis, scRNA‐seq was subsequently applied to rice, maize, tomato, peanut, cabbage, poplar, and cotton. scRNA‐seq was instrumental in elucidating plant responses to heat stress, uncovering spatially resolved molecular adaptations. Notable applications included a single‐cell atlas of heat‐stressed cabbage leaves that revealed subgenome dominance dynamics (Sun et al. 2022), and a cotton anther transcriptome that identified heat‐induced male sterility mechanisms at the tetrad stage (Li, Ma, et al. 2024). However, protoplast‐based scRNA‐seq faced challenges such as variable cell‐wall digestion efficiency and the potential impact of protoplast preparation on authentic gene expression (Shulse et al. 2019). Consequently, single‐nucleus RNA sequencing (snRNA‐seq) was developed as an alternative approach that removed cells with high expression of cytoplasmic organelle‐associated genes and avoided artefacts introduced by enzymatic digestion (Deal and Henikoff 2010). This method was successfully applied to cell‐type‐specific transcriptome analyses in rice roots, Arabidopsis embryos, and tomato fruits (Farmer et al. 2021; Palovaara and Weijers 2019; Reynoso et al. 2018). Nevertheless, single‐cell or single‐nucleus studies in non‐model plants and under abiotic stress responses remained limited. Notably, no systematic cell‐type identification or expression profiling had been conducted in potato leaves.

Although scRNA‐seq/snRNA‐seq have revealed cell type‐specific stress responses in model plants such as Arabidopsis and rice, whether these findings fully represent potato has remained a critical knowledge gap. Therefore, in this study, we employed snRNA‐seq to characterise, for the first time, the single‐nucleus transcriptomic landscape of potato under both normal growth and heat stress conditions. By comparing samples exposed to different temperature conditions, we identified distinct cell clusters and their marker genes within potato leaves. Pseudo‐time analysis reconstructed the development and differentiation trajectories of various cell types. The results indicated that the PIF4, a key regulator of heat‐stress response, enhanced plant heat tolerance by modulating ROS accumulation. This study established an essential foundation for elucidating cell type‐specific regulatory mechanisms underlying potato responses to heat stress.

## 2 Results

### 2.1 Construction of A Potato Leaf Cell Atlas

The experiment included two treatments: control (CK; 22°C day/18°C night) and heat stress (35°C day/28°C night for 24 h), each comprising three biological replicates. Preliminary quality control using Cell Ranger yield 50 126 and 39 922 high‐quality nuclei from the CK and heat‐stress samples, respectively. After removing duplicates, low‐quality nuclei, and lowly expressed genes, 42 673 and 34 671 high‐quality nuclei were retained for the CK and heat‐stress samples, respectively. In total, the CK and heat‐stress samples generated 1 131 276 404 and 1 078 177 573 raw reads, respectively, with valid barcode ratios of 92.90% and 93.53%. Of these reads, 86.77% and 86.83% were uniquely and confidently mapped to the potato reference genome. The snRNA‐seq data identified 29 676 and 29 864 expressed genes in the CK and heat‐stress samples, respectively. The mean number of genes detected per nucleus was 1497 for CK and 1649 for heat stress, and these nuclei were used for downstream analysis. The average number of unique molecular identifiers (UMIs) per nucleus was 1931 in CK and 2217 under heat stress (Table S1). Nuclei from independent replicates were integrated and pooled for subsequent analysis, demonstrating high concordance among replicates.

**FIGURE 1:**
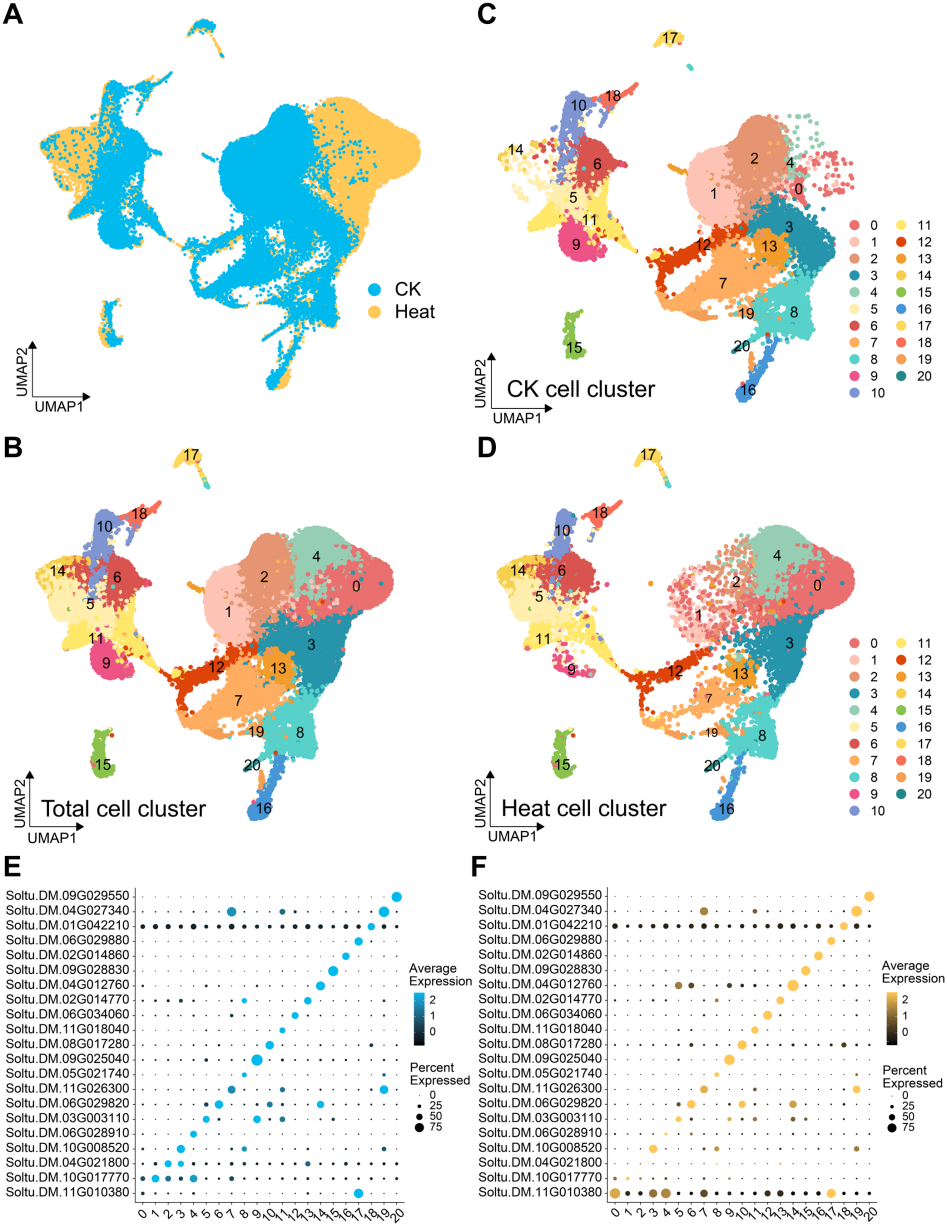
Single‐nucleus transcriptome sequencing atlas of potato leaves. (A) UMAP plot illustrated leaf cell clusters of CK and heat‐stressed seedlings. Each dot represented a cell. The cells from CK and heat‐stressed seedlings were represented in blue and yellow colour, respectively. (B) 21 colours represented 21 distinct cell clusters identified snRNA‐seq. (C, D) Leaf cell clusters of CK and heat‐stressed samples, respectively. (E, F) Expression patterns of representative genes for per cell cluster in CK and heat‐stress samples, respectively. Bubble plots and colour gradients illustrates the expression pattern and distribution of these representative genes. Standardised average expression levels using the *Z*‐Score method. UMAP better preserved the global structure of the data, so its results were selected for the main analysis.

To characterise the cellular composition of potato leaf cells, all high‐quality nuclei were subjected to clustering and dimensionality reduction. The nuclei were clustered into 21 distinct groups and visualised with t‐distributed stochastic neighbour embedding (t‐SNE) and uniform manifold approximation and projection (UMAP). Based on correlation analyses, UMAP provided a more biologically meaningful representation of cellular organisation and cluster distribution (Figure 1A–D, Figure S1A–E). Dimensionality reduction of the CK and heat‐stress samples revealed that cell clusters showed greater dispersion under heat stress. The expression patterns of the top differentially expressed gene (DEG) markers for each cluster (genes exhibiting similar expression patterns across multiple clusters) were altered under heat stress (Figure 1; Figure S1). These findings suggested that some clusters might originate from the same cell type and indicated that heat stress induced greater transcriptome heterogeneity within potato leaf cells. Collectively, these analyses enabled the reconstruction of a single‐nucleus atlas of potato leaf cells, revealing 21 distinct transcriptional clusters.

### 2.2 Identification of Major Cell Types in Potato Leaves

**FIGURE 2:**
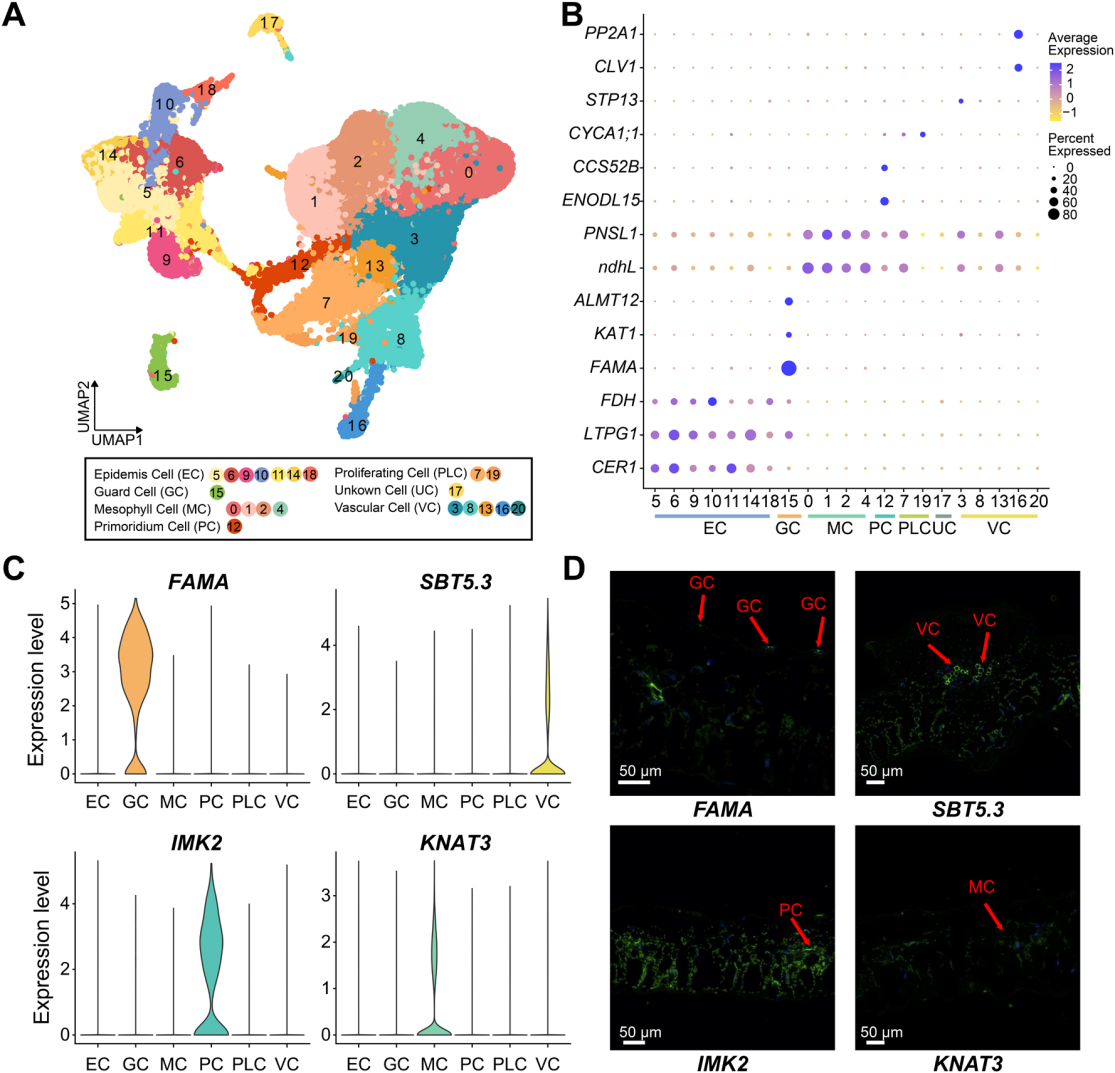
Identification of cell types in potato leaves. (A) Cell type delineation of UMAP visualised cell clusters. Colour, cell clusters. (B) Expression patterns and distribution of reported cell type‐specific marker genes in seven cell types. Standardised average expression levels using the *Z*‐Score method. Resolution = 0.5. (C) Violin plots show the expression distribution of four marker genes for cells in the potato leaf. The violin plots indicate gene expression distributions. Width indicates data density. The embedded box shows the interquartile range with the median line. EC, epidermal cell; GC, guard cell; MC, mesophyll cell; PC, primordium cell; PLC, proliferating cell; VC, vascular cell. (D) RNA in situ hybridization assays for four clusters‐specific genes in the potato leaf. In the in situ hybridization antisense images, the identified cell types (shown at the bottom) are indicated by red arrowheads. Bars, 50 μm.

To annotate potato leaf cell clusters, we performed ortholog‐based cell type prediction using known marker genes from Arabidopsis, other plant species, and the scPlant database (He et al. 2024). The 21 cell clusters identified by snRNA‐seq were assigned to six major types (Figure 2A; Table S2): epidermal cells (EC, cluster 5, 6, 9–11, 14, 18), guard cells (GC; cluster 15), mesophyll cells (MC; cluster 0–2, 4), primordial cells (PC; cluster 12), proliferating cells (PLC; cluster 7, 19), and vascular cells (VC; cluster 3, 8, 13, 16, 20). MCs, the most abundant type (45.49%), showed enrichment of genes such as *ndhL* and *PNSL1*, consistent with their photosynthetic function (Ifuku et al. 2011). ECs were characterised by marker genes including *CER1*, *FDH* and *LTPG1* (Debono et al. 2009; Guo et al. 2024). GCs specifically expressed *KAT1*, *FAMA*, and *ALMT12* (Liu et al. 2020). PCs expressed *ENODL15* (Smolarkiewicz et al. 2014) and *FZR3/CCS52B*, while PLCs were characterised by *CYCA1;1*, indicating active cell division (Zhang et al. 2021). Collectively, these markers outlined the regulatory network governing cell differentiation and proliferation during leaf development. VCs displayed co‐expression of *STP13*, *CLV1*, and *PP2A1*, demonstrating their roles in metabolite transport and intercellular signalling (Hirakawa et al. 2011; Kim et al. 2021) (Figure 2B, Table S3). Collectively, the systematic identification of these marker genes provided a robust foundation for the precise classification of potato leaf cell types. Additionally, we selected representative DEGs: *FAMA* (Soltu.DM.09G028830, GCs), *SBT5.3* (Soltu.DM.02G014770, VCs), *IMK2* (Soltu.DM.09G009290, PCs), and *KNAT3* (Soltu.DM.08G009980, MCs). The expression patterns of these selected DEGs, assessed by RNA in situ hybridization, were consistent with their predicted cell types (Figure 2C,D). These results provided a reliable basis for precise cell type identification in potato leaves.

**FIGURE 3:**
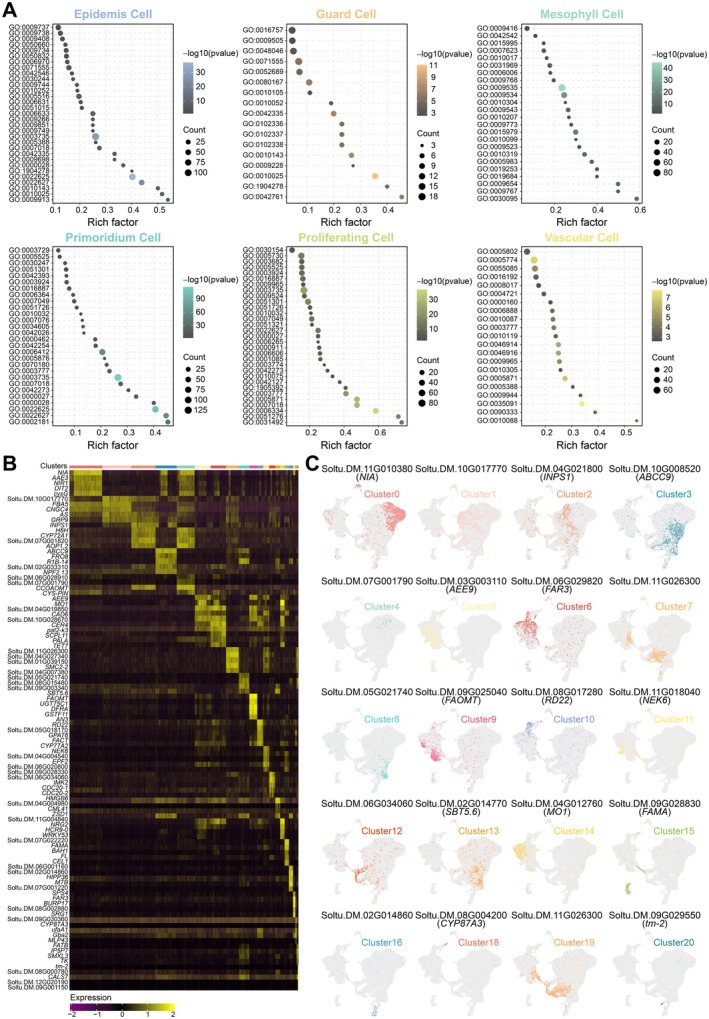
Novel leaf cell type‐specific marker genes in potato. (A) Go enrichment analysis of cell type‐specific DEGs. Colour, cell types. (B) Expression patterns of the top 5 marker genes for each cluster. Standardised average expression levels using the *Z*‐Score method. (C) UMAP visualisation of representative marker gene expression across clusters. Each dot represents a single cell, with colour intensity indicating gene expression levels. Grey denotes no expression; coloured shading indicates positive expression. Clusters are coloured according to their assigned identities.

Gene Ontology (GO) enrichment analysis of cell type‐specific differentially expressed genes (DEGs) revealed significant enrichment in distinct biological processes. EC‐DEGs were enriched in fatty acid metabolism and lipid transport, supporting cuticle formation. GC‐DEGs were linked to thiamine biosynthesis and ethylene signalling, reflecting functions in energy metabolism and stomatal regulation. MC‐DEGs were associated with photosynthesis‐related processes, including photosynthetic membrane assembly and starch catabolism. DEGs from PCs and PLCs were enriched in cell cycle regulation and meristem maintenance, consistent with their proliferative activity. VC‐DEGs were involved in microtubule motor activity and hormone transport, indicating functions in intracellular movement and signalling (Figure 3A; Table S4). These cell type‐specific GO profiles validated the accuracy of our ortholog‐based marker identification strategy using the scPlant database, providing a reliable resource for functional genomics research in potato.

### 2.3 Characterisation Analysis of Novel Leaf Cell Type‐Specific Marker Genes in Potato

While plant single‐cell databases (e.g., for Arabidopsis, rice, maize, cotton, and peanut) contain marker genes and expression profiles across cell types, we observed that these markers exhibited limited specificity and inconsistent expression patterns in potato leaf cell types. Therefore, it was essential to identify genes specifically expressed in distinct potato leaf cell types.

We performed gene expression analysis across seven cell clusters (ECs, GCs, MCs, PCs, PLCs, VCs, and an unknown cluster) to identify potential cell‐type‐specific marker genes. Two thresholds were applied: (1) higher average expression in the target clusters compared to all other clusters, and (2) expression in ≥ 25% of cells within the target clusters and < 25% of cells in non‐target clusters. Genes meeting these thresholds, combined with an adjusted *p*‐value ≤ 0.01 and |log2 fold change (FC)| ≥ 0.5, were identified as highly expressed and cell‐type‐specific. This approach identified 467 EC‐specific, 44 GC‐specific, 194 MC‐specific, 54 PC‐specific, 234 PLC‐specific, 454 VC‐specific, and 249 unknown cluster‐specific marker genes (Table S5). Identified markers included genes with known functions, such as: the wax biosynthesis gene *KCS3* (Soltu.DM.11G026450) in ECs; the guard cell differentiation‐promoting gene *FAMA* (Soltu.DM.09G028830, Soltu.DM.05G023840) and a putative Na^+^/H^+^ antiporter gene *CHX18* (Soltu.DM.08G028550) in GCs; the sucrose phosphate synthase gene *SPS* (Soltu.DM.07G003160) in MCs; the mitotic cell cycle regulator *SCL28* (Soltu.DM.08G027050) in PCs; and the copper chaperone gene *CCH* (Soltu.DM.11G006190) and phloem protein gene *PP2A1* (Soltu.DM.02G011940) in VCs. Numerous uncharacterized genes were also identified. The top 10 most highly expressed marker genes per cell type were further analysed to validate their cell‐type specificity (Table S5).

To elucidate cluster‐specific molecular functions, the top 5 most highly expressed genes in each cluster were subjected to expression pattern analysis and GO/Kyoto Encyclopedia of Genes and Genomes (KEGG) functional annotation, enabling the systematic identification of representative marker genes (Figure 3B,C). KEGG pathway enrichment analysis indicated that the significantly enriched pathways in major cell clusters were predominantly associated with metabolic and energy‐related processes (Table S6). These marker genes demonstrated robust cell‐type specificity and will facilitate precise cell type classification in potato.

### 2.4 Heat Stress Promotes the Differentiation of Leaf Cells Along Pseudo‐Time Trajectories

**FIGURE 4:**
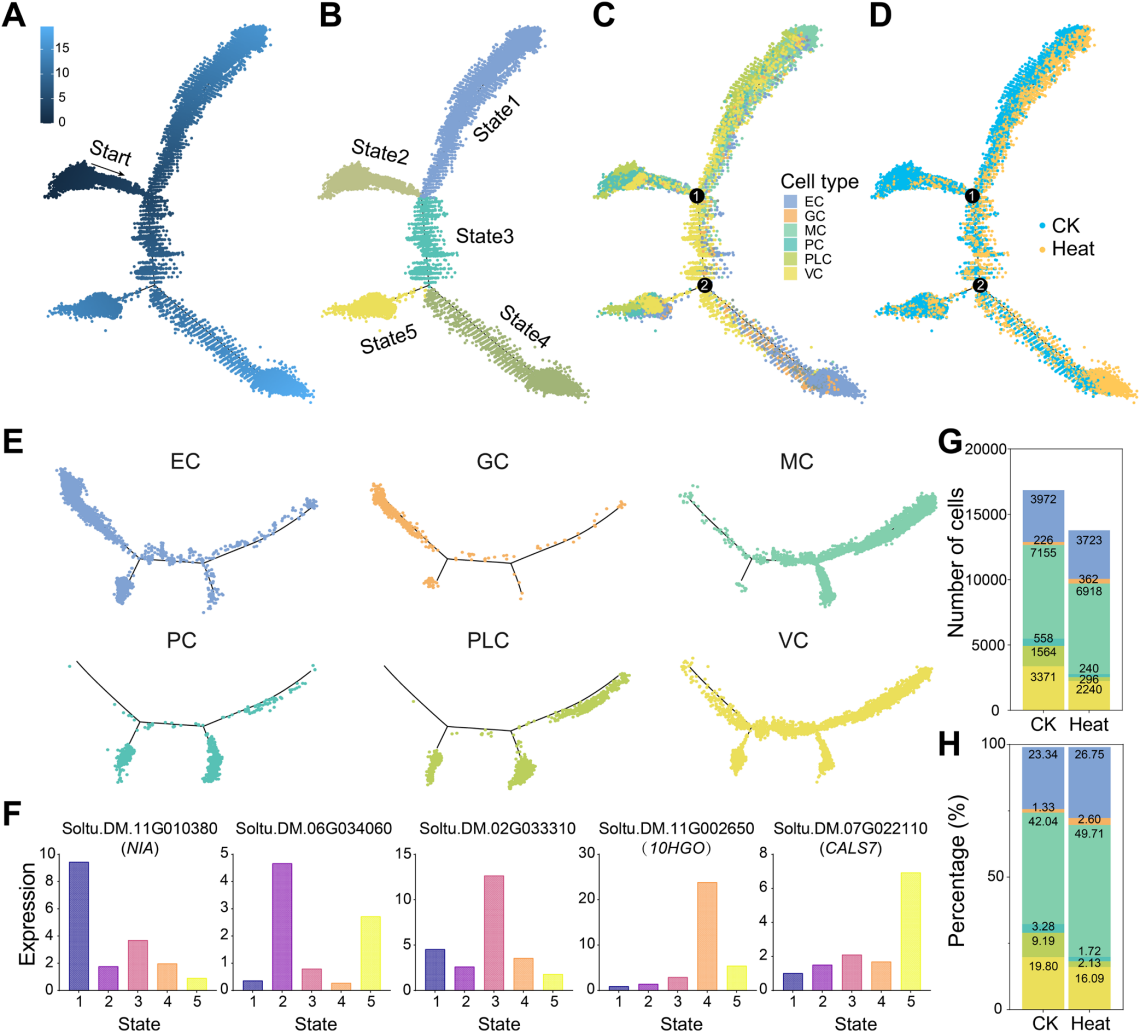
Pseudo‐time trajectory analysis of cell types in leaves of CK and heat‐stress potato seedlings. (A–D) Single‐cell pseudo‐time trajectory plots. Each dot represents an individual cell. The trajectory is visualised by (A) pseudo‐time ordering, (B) inferred cell state, (C) cell type identity, and (D) treatment condition. (E) Trajectory distribution of six cell types along pseudo‐time. Colours, cell types. (F) Bar plot of representative gene expression across cell states. (G, H) Separate bar plots of cell number and proportion for six cell types under CK and heat‐stress conditions.

To analyse the developmental dynamics of potato leaf cells, this study employed pseudo‐time trajectory analysis using Monocle 2, involving 30 937 total cells (CK: 17019; heat stress: 13918) (Figure 4G,H; Table S7). Cells were classified into five states (State 1–5), with State 2 identified as the root of the trajectory and enriched in PLCs and PCs; State 1 predominantly comprised MCs, States 3 and 5 were enriched in VCs, and State 4 comprised ECs and GCs (Figure 4A–E). Five key marker genes (*NIA*, Soltu.DM.06G034060, Soltu.DM.02G033310, *10HGO*, and *CALS7*) were identified along the trajectories, thereby enabling the discrimination of distinct developmental states (Figure 4F). The distribution pattern of the unknown cell type closely resembled that of GCs, suggesting that they may belong to the same or developmentally related cell types. Additionally, two branching nodes were observed within the trajectory: Node 1 was associated with 9527 specific‐DEGs, enriched in processes such as primary metabolism, plastid metabolism, and protein transport, and Node 2 included 420 specific‐DEGs primarily involved in pathways such as gene expression and chromosome organisation (Figure 4D). Node 1 corresponds to the differentiation of PLCs toward MCs, while Node 2 is associated with EC aggregation, which becomes more pronounced under elevated temperatures, suggesting that genes associated with Node 2 may play a critical role in thermal responses (Table S8).

Heat stress severely reduces yield in cool‐weather crops such as potato. However, the extent to which distinct leaf cell types exhibited divergent responses under heat stress remained unclear. Compared with the CK samples, the relative proportions of ECs (26.75%), MCs (49.71%), and GCs (2.60%) increased, while those of meristematic and proliferative cells decreased (Figure 4G,H). Despite the lower total cell count in heat‐stress samples, the numbers of ECs, MCs, and GCs at the trajectory endpoints still exceeded those in CK. These findings imply that heat stress may act as a catalyst, promoting differentiation along the developmental trajectory of potato leaf cells. To further validate this inference, we conducted a re‐clustering analysis on PCs and PLCs (Figure S2A,B). Under heat stress, both the absolute number and relative proportion of these two cell populations were significantly reduced. Notably, the marker genes of subcluster 10 overlapped significantly (9/10) with the top 10 marker genes identified in leaves. However, these overlapping genes were predominantly highly expressed in VCs following heat stress, followed by ECs and MCs (Figure S2C). Based on the trajectory distributions (VCs situated in the transition zone between nodes 1 and 2, MCs concentrated on node 1 branches), this subcluster was hypothesised to represent a group associated with vascular cell fate determination. The genes of this subcluster activate under heat stress, suggesting that VCs play a crucial role in heat stress responses. 82 genes were uniquely identified as significantly upregulated under heat stress (Figure S2D, Table S9). They were upregulated in ECs, MCs, and VCs and enriched in pathways such as stress response, nitrogen metabolism, and photosynthesis. These findings suggested that potato leaves may enhance adaptability to heat stress by accelerating the differentiation of meristematic cells into functional cells, through increased photosynthetic and metabolic activity (Figure S2E; Table S10).

### 2.5 Identification of Core TFs Regulating Leaf Cell Response to Heat Stress

**FIGURE 5:**
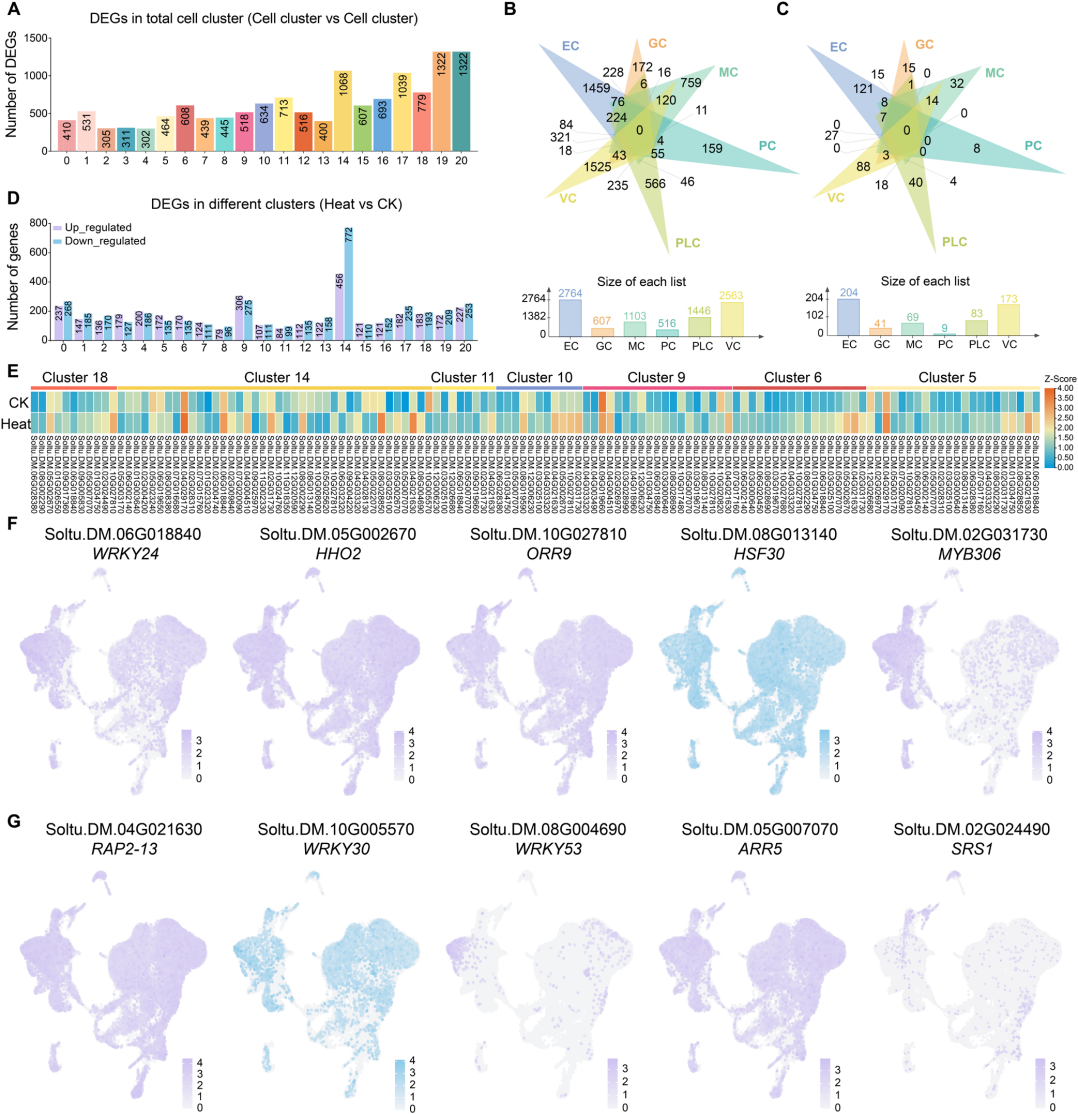
Details of DEGs in various cell clusters and cell differentiation process. (A) The number of DEGs in each cell cluster. (B, C) Venn diagram of core and specific DEGs and TFs in all cell types. (D) Bar plots of up‐ and down‐regulated DEGs in each cell cluster. (E) The heat map represented the average expression level of core‐TFs in each cell cluster under CK and heat stress conditions, based on data from three biological replicates. (F, G) Single‐nucleus expression patterns of DETFs. Purple, up‐regulation; blue, down‐regulation. Darker colours denote higher expression levels.

To identify core genes within distinct potato leaf cell subclusters, differential expression analysis was performed. The number of upregulated DEGs per cell cluster ranged from 302 to 1322. Venn diagram analysis revealed overlapping DEGs across clusters, enabling the identification of shared core genes (Figure 5A,B). Several marker genes with specific expression patterns were identified within ECs, such as *HSFA2* (Soltu.DM.08G013140), *HSFA3* (Soltu.DM.09G004220), *MOM1* (Soltu.DM.10G005910), and *DnaJ* (Soltu.DM.04G001130). These genes function in heat‐stress response across species, suggesting that ECs actively contribute to heat stress adaptation (Figure S3A). Given the crucial role of transcription factors (TFs) in regulating gene expression, 427 differentially expressed TFs (DETFs) were identified across all clusters (Figure 5C). KEGG annotation revealed that these DETFs were primarily involved in signalling pathways (including ethylene and auxin signalling), signal transduction, environmental adaptation, and genetic information processing (Table S11).

Comparative analysis of DEGs between the CK and heat stress conditions effectively reduced the number of candidate DEGs and TFs. This comparative profiling of samples under CK and heat stress facilitated the identification of stress‐upregulated and stress‐downregulated DEGs (Tables S12, S13). Among EC clusters, cluster 14 exhibited the highest number of stress‐responsive DEGs, followed by cluster 9 (Figure 5D). Within these two clusters, 62 DETFs were identified as associated with stress response and plant hormone signal transduction (Figure 5E; Table S14). Furthermore, systematic integration of fold‐change values, adjusted *p‐*values, and UMAP expression patterns identified 10 candidate TFs. Expression profiling across individual nuclei revealed distinct regulatory patterns for these TFs. Notably, TFs *WRKY24*, *HHO2*, *ORR9*, *MYB306*, *RAP2‐13*, *WRKY53*, *ARR5*, and *SRS1* exhibited marked upregulation in most nuclei under heat stress. In contrast, *HSF30* and *WRKY30* were predominantly expressed under CK conditions (Figure 5F,G; Table S13). These core TFs represent valuable genetic candidates for uncovering key regulators of potato leaf cell differentiation under variable thermal conditions.

**FIGURE 6:**
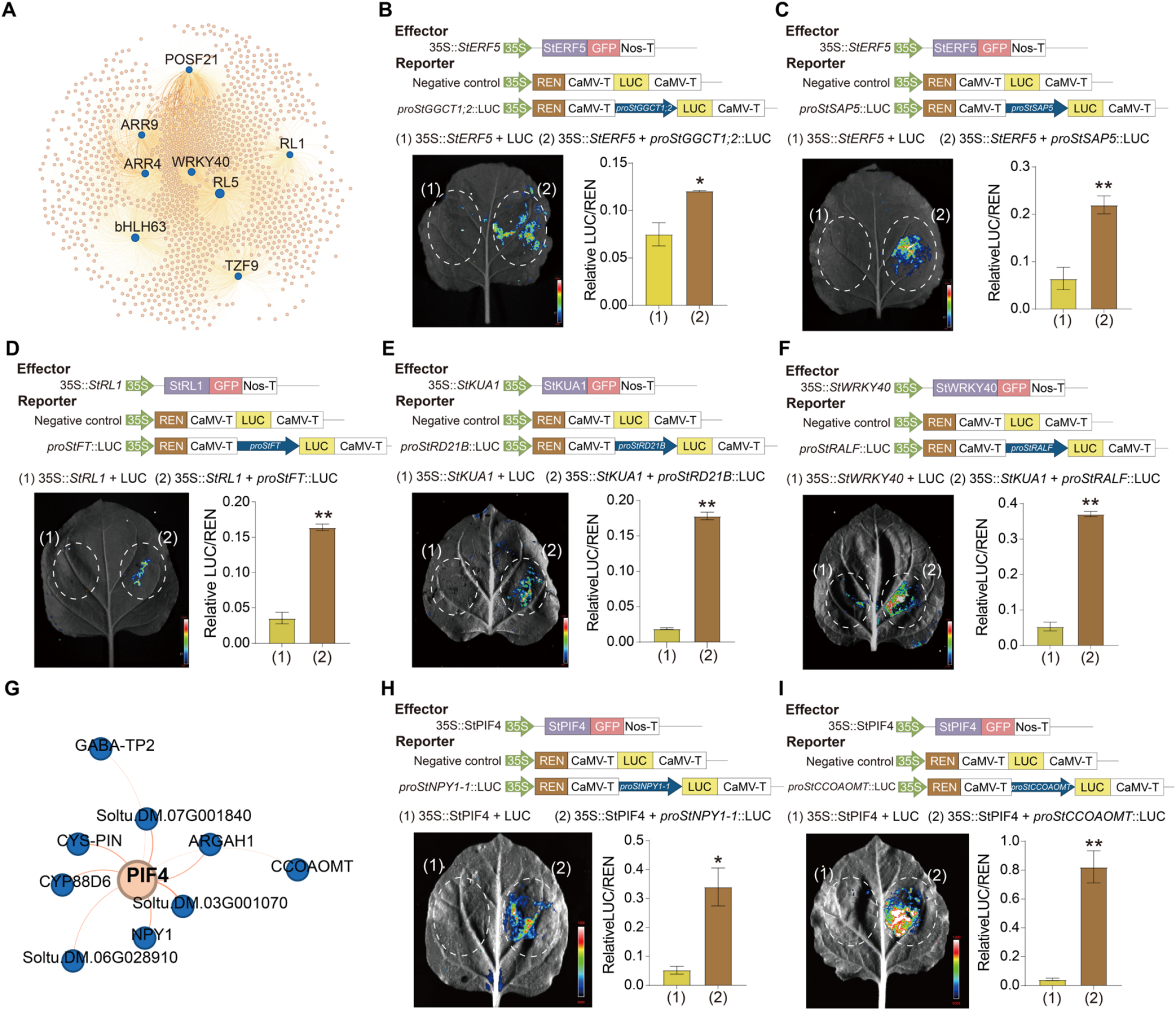
Transcriptional co‐expression network of potato leaves in response to heat stress at single‐cell resolution. (A) Co‐expression network of WGCNA brown module core TFs. Blue dots: Candidate core TFs within the brown module; Orange dots: Corresponding co‐expressed genes of the candidate core TFs; Dot size: Degree; Edges: Linking candidate core TFs and their co‐expressed genes at either end, forming co‐expression gene pairs. As the weight increases, edges become thicker and darker in colour. (B–F) Interaction between TFs and predicted target genes. *StERF5*, *StRL1*, *StKUA1* and *StWRKY40* were cloned and constructed into a pCAMBIA1300‐GFP expression vector. Upstream sequences (2000 bp upstream of ATG) of *proStGGCT1;2*, *proStSAP5*, *proStFT*, *proStRD21B* and *proStRALF*, containing various motifs, were cloned and inserted into the pGreenII‐0800‐LUC vector. Fluorescence signals were observed 48 h later. Red represented the maximum value and blue represented the minimum value. Enzyme activity was measured after punch‐collecting uniform‐sized tobacco leaves, *n* = 3. Significance analysis was performed using Student's *t*‐test. *p‐value < 0.05; **p‐value < 0.01. (G) Local co‐expression networks between *PIF4* and core genes. Blue dots, core genes; Orange dots, co‐expression genes; dot size, degree; Edges: Linking candidate core gene and their co‐expressed genes at either end, forming co‐expression gene pairs. As the weight increases, edges become thicker and darker in colour. (H, I) Dual‐luciferase assay of transcription factors and predicted target genes; StPIF4‐*StNPY1‐1* (H), StPIF4‐*StCCOAOMT* (I), The promoter is located 2000 bp upstream of ATG. *p‐value < 0.05; **p‐value < 0.01.

Pseudo‐time trajectory analysis identified 7171 differentiation‐related DEGs, including 203 heat‐responsive TFs (Figure S3B; Table S15). Furthermore, analysis of 11 107 DEGs associated with differentiation states identified 319 key heat‐responsive TFs (Figure S3C; Table S16). Notably, 3844 DEGs and 143 heat‐responsive TFs were identified at Node II, highlighting lineage‐specific differentiation across environmental conditions (Figure S3D; Table S17). Through multi‐dimensional identification, 13 core differentiation‐related TFs were ultimately identified as potential bridges between development and heat stress response (Figure S3E). Integration of bulk RNA‐seq data enabled a holistic exploration of gene‐expression complexity and dynamics, and we ultimately identified 32 common TFs, including 12 core single‐nucleus TFs (Figure S4A,B; Tables S18, S19). Co‐expression analysis revealed that eight regulators (including *POSF21*, *WRKY40* and *ARR9*) clustered in the brown module with coordinated expression under heat stress (Figure S4C,D). The co‐localization of these key transcription factors and signalling components, which are known to function in heat, abscisic acid, and cytokinin responses, suggested that the brown module represents a core regulatory network for integrating multiple stress signals and orchestrating transcriptional reprogramming under heat stress. The *NAC091* co‐expression network included classical heat‐responsive genes such as *HSFB2b* and *HSP70* (Ikeda et al. 2011; Xu et al. 2024). These findings collectively indicate that the 12 core TFs likely play central roles in orchestrating thermal adaptation in potato leaves, thereby establishing key genetic targets for elucidating cellular heat response mechanisms (Figure 6A; Figure S4E–I; Table S20).

To validate the reliability of the predicted regulatory network, five TF‐target gene pairs were selected for experimental verification. These pairs involved five TFs and their seven corresponding target genes. Dual‐luciferase assays revealed that *StERF5* significantly activated the transcription of both *StGGCT1.2* and *StSAP5*. The relative LUC/REN activity ratios further confirmed that *StERF5*'s transcriptional activity was markedly enhanced upon co‐expression with *StGGCT1.2* and *StSAP5*, respectively (Figure 6B,C). Co‐expression of *StRL1* with *StFT*, *StKUA1* with *StRD21B*, and *StWRKY40* with *StRALF* significantly activated the promoters of target genes, as evidenced by increased luciferase activity (Figure 6D–F). Collectively, these results support the predicted regulatory relationships between StERF5‐*StGGCT1;2*, StERF5‐*StSAP5*, StRL1‐*StFT*, StKUA1‐*StRD21B*, StWRKY40‐*StRALF*, StPIF4‐*StNPY1‐1*, and StPIF4‐*StCCoAOMT*, thereby reinforcing the accuracy and biological relevance of the sequencing‐based network predictions.

### 2.6 *StPIF4* Enhances Thermotolerance Under Heat Stress in Potato

**FIGURE 7:**
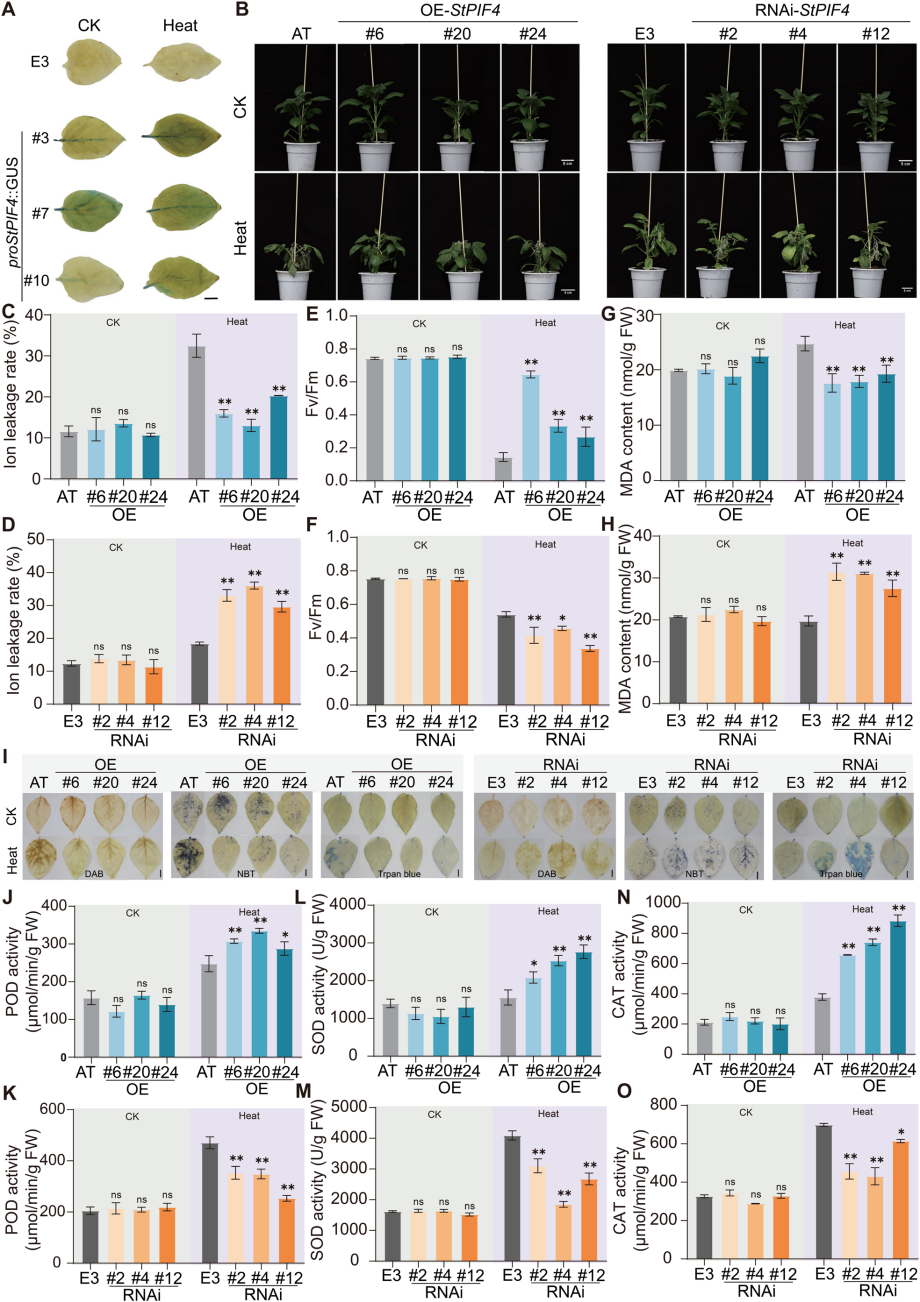
*StPIF4* enhances thermotolerance under heat stress in potato. (A) GUS staining assay of *proStPIF4*::GUS transgenic potato lines under heat stress (42°C), *n* = 3. Scale bar = 1 cm. E3: ‘E potato 3’ wild‐type cultivar. (B) Phenotypes of wild‐type and *StPIF4* transgenic plants under CK and heat stress (42°C) for 48 h, *n* = 15. Scale bar = 5 cm. (C, D) Ion leakage rates in leaves of wild‐type (AT) and *StPIF4* overexpression lines (C), and wild‐type (E3) and RNAi lines (D), under CK and heat stress (42°C) for 48 h. (E, F) Fv/fm values of wild‐type (AT) and overexpression lines (E), and wild‐type (E3) and RNAi lines (F) under CK and heat stress (42°C) for 48 h. (G, H) MDA content in wild‐type (AT) and overexpression lines (G), and wild‐type (E3) and RNAi lines (H) CK and heat stress (42°C) for 48 h. (I) Histochemical staining of ROS and cell death using DAB, NBT, and trypan blue in wild‐type and *StPIF4* transgenic plants under CK and heat stress (42°C) for 48 h. Scale bar = 1 cm. (J, K) POD activity in wild‐type (AT) and *StPIF4* overexpression lines (J), and wild‐type (E3) and RNAi lines (K) under CK and heat stress (42°C) for 48 h. (L, M) SOD activity in wild‐type (AT) and overexpression lines (L), and wild‐type (E3) and RNAi lines (M) under CK and heat stress (42°C) for 48 h. (N, O) CAT activity in wild‐type (AT) and overexpression lines (N), and wild‐type (E3) and RNAi lines (O) under CK and heat stress (42°C) for 48 h. Data were presented as mean ± SEM (*n* = 3). Significant differences between transgenic lines and the corresponding wild‐type under the same condition were determined by two‐way ANOVA, *p‐value < 0.05; **p‐value < 0.01; ns, non‐significant.

Notably, within the co‐expression network of the turquoise module, multiple core genes formed co‐expression pairs with *StPIF4* (Figure 6G; Figure S4J–L). To further validate the reliability of the network, we detected the transcriptional levels of the *StNPY1* within the network through qRT‐PCR. The results showed that *StNPY1* expression levels were significantly upregulated in the OE‐*StPIF4* transgenic lines; conversely, its expression levels were significantly downregulated in the RNAi‐*StPIF4* transgenic lines (Figure S4M,N). Our result demonstrated that *StPIF4* significantly activated the transcription of *StNPY1‐1* and *StCCoAOMT* (Figure 6H,I). Among these, *CCoAOMT* was found to regulate lignin and anthocyanin synthesis, thereby playing a functional role in abiotic stress responses (Du et al. 2025). These results imply that *StPIF4* functions as a core regulator to directly activate stress‐responsive genes, thereby enhancing stress tolerance. To investigate the potential role of *StPIF4* in the response to heat stress, a *proStPIF4*::GUS reporter construct was constructed and introduced into potato via Agrobacterium‐mediated transformation (Figure S5A). Following 24 h exposure to 42°C, transgenic plant leaves exhibited a pronounced increase in GUS histochemical staining compared to the CK group (Figure 7A; Figure S5B). These observations indicated that the *StPIF4* promoter was activated by heat stress and supported a role for *StPIF4* in the heat stress response pathway.

To further explore the biological function of *StPIF4* under heat stress, three overexpression (OE) lines (OE‐6, OE‐20, OE‐24) and three RNA interference (RNAi) lines (RNAi‐2, RNAi‐4, RNAi‐12) were generated (Figure S5C,D). After 48 h of exposure to 42°C, although clear differences among genotypes were evident. The *StPIF4*‐OE lines displayed significantly reduced wilting and enhanced thermotolerance compared to the wild‐type (WT) (Figure 7B). In contrast, the RNAi lines exhibited more pronounced wilting and reduced heat tolerance (Figure 7B). Under control condition, no significant differences in relative electrolyte leakage were observed among the genotypes (Figure 7C,D). Under heat stress, ion leakage increased in all genotypes; however, it was significantly lower in *StPIF4*‐OE plants compared to the WT (Figure 7C). In contrast, RNAi lines showed markedly higher ion leakage than the WT (Figure 7D). The maximum quantum efficiency of photosystem II (Fv/fm) decreased under heat stress in all genotypes (Figure 7E,F). However, OE lines maintained significantly higher Fv/fm values than the AT (Figure 7E), whereas RNAi lines exhibited substantially lower values than E3, indicating exacerbated photoinhibition (Figure 7F). Similarly, malondialdehyde (MDA) content showed no significant differences among genotypes under control condition; however, under heat stress, it was markedly lower in overexpression lines and elevated in RNAi lines compared to their respective WT (Figure 7G,H). Collectively, these results demonstrated that *StPIF4* was a positive regulator of heat‐stress response pathway in potato.

To investigate the role of *StPIF4* in regulating ROS metabolism under heat stress, histochemical staining with diaminobenzidine (DAB), nitrotetrazolium blue chloride (NBT), and trypan blue was performed on leaves of WT and transgenic plants under CK and heat stress. The results showed no significant differences in staining intensity among genotypes under control conditions (Figure 7I; Figure S5E,F). After heat stress, all lines exhibited increased staining intensity; however, the *StPIF4* overexpression lines showed lighter DAB, NBT, and trypan blue staining compared to the WT, while the RNAi lines displayed more intense staining (Figure 7I; Figure S5E,F). Enzyme activity assays revealed no notable differences in peroxidase (POD), superoxide dismutase (SOD), and catalase (CAT) activities between WT and transgenic plants under control conditions (Figure 7J–O). However, under heat stress, the activities of all three enzymes changed significantly across genotypes (Figure 7J–O). Specifically, the *StPIF4* OE lines exhibited significantly higher POD (Figure 7J), SOD (Figure 7L), and CAT (Figure 7N) activities than the WT, whereas the RNAi lines showed significantly lower activities (Figure 7K,M,O). Collectively, these results demonstrated that *StPIF4* enhanced thermotolerance in potato by positively modulating the antioxidant enzyme system, thereby mitigating heat‐induced oxidative damage.

In Arabidopsis, PIF4 responds to heat stress by directly binding to *HSFA2* and activating its expression (Yang et al. 2022). Based on the identification of *StHSFA2* as an EC marker gene in this study (Figure S3A), we investigated the regulatory relationship between *StPIF4* and *StHSFA2* to further elucidate the *StPIF4*‐mediated regulatory pathway under heat stress in potato. qRT‐PCR results showed that *StHSFA2* expression was upregulated in the OE‐*StPIF4* line and downregulated in the RNAi‐*StPIF4* line under ambient temperature conditions (Figure S6A). Following heat stress, *StHSFA2* was significantly induced in all genotypes. The induction level in the overexpressing line was markedly higher than that in the wild‐type, while the RNAi line exhibited lower induction than the wild‐type (Figure S6A). This indicates that *StHSFA2* expression is positively regulated by StPIF4, suggesting StPIF4 may enhance potato heat tolerance by activating *StHSFA2* transcription. LUC assays demonstrated that StPIF4 binds to the *StHSFA2* promoter and enhances its transcriptional activity (Figure S6B,C). Furthermore, the specificity of direct binding between StPIF4 and the E‐box element in the *StHSFA2* promoter was confirmed by an electrophoretic mobility shift assay (EMSA) (Figure S6D). These results suggest that *StPIF4* enhances heat tolerance in potato through the transcriptional activation of *StHSFA2*.

## 3 Discussion

Heat stress represents a significant threat to global food security, severely impeding potato growth, yield, and quality (Zhao et al. 2017). In the field of plant single‐cell biology, research on heat stress responses remains underdeveloped. The direct application of existing research data to potato is unlikely to be sufficient for comprehensively elucidating their distinctive heat stress response mechanisms. To address this gap, this study presents the first cell‐type‐specific atlas of potato leaf cells under heat stress, systematically revealing divergent response mechanisms across distinct cell types under elevated temperature conditions. These findings provide a fundamental framework for elucidating molecular mechanisms of plant stress adaptation and advance our understanding of leaf function in potato and related crops at nuclear resolution. Consequently, this work advances the plant single‐cell stress research system from model plants to potato, establishing a novel framework for elucidating the thermal response mechanisms of genetically complex crops. Furthermore, it offers valuable methodological insights and a theoretical foundation for future research on other non‐model crops.

In this study, through a comprehensive analysis of cell‐type‐specific marker genes, we classified all nuclei into 21 clusters, which were subsequently grouped into six major cell types, establishing a foundation for investigating leaf development in potato (Figures 1 and 2A). Cross‐species comparisons revealed that specific gene expression patterns were highly conserved between potato and Arabidopsis, as well as other dicotyledonous plants, with multiple marker genes (e.g., *FAMA*, *ENODL15*, *STP13*, *CLV1*, and *PP2A1*) exhibiting consistent cell‐type‐specific expression profiles (Figure 2B; Table S3) (He et al. 2024; Hirakawa et al. 2011; Kim et al. 2021; Smolarkiewicz et al. 2014; Tenorio Berrío et al. 2022) (Figure 2B; Table S3). Notably, the potato single‐cell atlas also displayed species‐specific heterogeneity: while RBCSs genes are widely recognised as MC markers in Arabidopsis, peanut, and cabbage, no such specificity occurred in potato (Deng et al. 2024; Sun et al. 2022). The single‐cell profiles of potato shoot apex and stolon tips shared similar cell‐type compositions with leaves, indicating a conserved cellular framework across tissues (Guo et al. 2025). Comparisons with Arabidopsis confirmed species‐specific transcriptional differences at single‐cell resolution, suggesting that, despite both being dicotyledonous species, potato possesses a distinct developmental program linked to tuber formation (Figure 2). This divergence may originate from interspecies genetic divergence, functional differentiation of tissues/organs, or technical differences in experimental protocols. To address knowledge gaps in cross‐species cellular heterogeneity, we identified 1696 novel potato leaf cell‐specific marker genes, providing critical molecular resources to decipher species‐specific mechanisms of leaf cell structure and function (Figure 3; Table S5).

Pseudo‐time trajectory analysis indicated that heat stress did not alter the fundamental developmental pathways of potato leaf cells but did influence gene‐expression dynamics across different cell populations (Figure 4E). This aligns with reports that heat stress preserves cellular identity while modifying expression levels of cell type‐specific genes (Sun et al. 2022). Following heat stress, the relative proportions of ECs, MCs, and GCs were increased. Among these, ECs harboured significantly more DEGs than other cell types, whereas PLCs and PCs exhibited fewer DEGs (Figures 4F,G and 5A,D). Notably, distinct cellular lineages across species exhibited markedly divergent patterns of response to heat stress. For example, the most pronounced responses were observed in the cortex cells of maize roots, the vascular cells of pearl millet leaves, the mesophyll cells of Chinese cabbage leaves, and the tapetum cells of cotton anthers (Jin et al. 2025; Li, Ma, et al. 2024; Sun et al. 2022; Wang et al. 2025). This diversity in response patterns provided compelling evidence for the species‐specific nature of plant heat stress adaptation. Furthermore, our analysis revealed that cell‐type‐specific expression changes predominantly occurred in differentiated cells, consistent with observations from abiotic stress studies of Arabidopsis roots, rice seedlings, and cabbage shoot apices (Cheng et al. 2012; Sun et al. 2022; Wang et al. 2021). Subpopulation analysis of PCs and PLCs further indicated that heat stress redirects meristematic cells toward VCs, ECs, and MCs lineage specification in potato leaves (Figure S2B,C). VCs were not the most transcriptionally altered cell type in this study, while they were implicated in the critical transition from progenitor cells to functional vascular cells. This suggests VCs play a key role in potato's heat stress response. Consistent with prior studies, we hypothesise that VCs play a fundamental role in plant heat stress responses, although their specific manifestations might vary across species and tissues. GO enrichment analysis of DEGs in ECs under heat stress showed significant enrichment in regulatory pathways (e.g., localization and transport regulation) and nucleotide/cofactor metabolism (Figure 3A). In contrast, VCs exhibited a bias toward entries related to cellular homeostasis and sulfur compound/amino acid metabolism (Figure 3A). While snRNA‐seq data cannot directly prove physical interactions, this functional enrichment pattern suggested a clear functional division of labour and collaborative intercellular cooperation. This provided a refined model for understanding multicellular coordination of stress responses. Additionally, it revealed that although different crops utilise distinct primary cell types, the core principle of functional specialisation and coordination of cell types remains conserved across species.

Comparative single‐cell transcriptomics across plant species revealed that rapid transcriptional reprogramming induced by heat stress exhibited cell‐type specificity. Similar to findings in maize roots and foxtail millet leaves, MCs of potato showed early induction of HSPs and reactive‐oxygen‐species scavengers, whereas epidermal and vascular tissues exhibited delayed activation of abscisic‐acid and ethylene‐signalling pathways (Jin et al. 2025; Wang et al. 2025). Pseudo‐time trajectories further revealed that stress‐responsive TFs such as *HSFA2* and *DREB2A* exhibited a spatiotemporal activation pattern progressing from photosynthetic to structural tissues, thereby unveiling a conserved regulatory architecture of plant HT response at the single‐cell level (Figure 4). The study also indicated that outermost cells responded more rapidly to external stress, consistent with reports that cortex cells expressed the highest number of heat‐shock‐specific genes in maize roots (Wang et al. 2025).

Previous studies have established that *PIF4* regulates thermomorphogenesis by activating auxin‐ and brassinosteroid‐biosynthesis genes and enhances thermotolerance in Arabidopsis by directly promoting heat‐shock protein expression (Koini et al. 2009; Xu and Zhu 2021; Yang et al. 2022). In Arabidopsis, PIF4 inhibits brassinosteroid synthesis under high‐temperature conditions by competing with BES1/BZR1 for binding to the BES1 homodimer, thereby regulating hypocotyl elongation (Martínez et al. 2018). The SPA1 protein directly phosphorylates PIF4 and affects its stability, participating in the regulation of thermomorphogenesis (Lee et al. 2020). Additionally, PIF4 interacts with the HOOKLESS protein to jointly regulate the thermomorphogenesis process (Jin et al. 2020). Nevertheless, there has been limited research on the function of *PIF4* under heat stress. Our study showed elevated GUS activity in transgenic potato lines harbouring *proStPIF4*::GUS after 42°C treatment (Figure 7A). *StPIF4*‐OE lines exhibited significantly enhanced thermotolerance, whereas RNAi lines displayed more severe wilting (Figure 7B). StPIF4 directly binds to and activates the promoters of *NPY1‐1* (Figure 6G), a key regulator of root gravitropism (Li et al. 2011), and *CCoAOMT* (Figure 6H), a gene involved in lignin and anthocyanin biosynthesis where *CCoAOMT1/7* enhance salt and drought tolerance (Du et al. 2025; Peng et al. 2024). Therefore, based on the known functions of its direct targets, we hypothesise that *StPIF4* might contribute to heat adaptation by coordinately influencing root architecture (via *NPY1‐1*) and stress‐responsive secondary metabolism (via *CCoAOMT*). Validating this hypothesis and the associated physiological outcomes in future root‐based experiments will be an important direction to elucidate the multifaceted roles of *StPIF4* under heat stress.

Under high‐temperature stress, the OE‐*StPIF4* lines exhibited higher antioxidant enzyme (POD, SOD, and CAT) activity and lower oxidative damage levels (Figure 7). Previous studies have indicated that *PIF4* is involved in the regulation of ROS homeostasis. In Arabidopsis, the *pif4* mutant accumulates higher levels of H_2_O_2_, and *WRKY33* modulates ROS homeostasis by regulating *PIF4* (Sun et al. 2020). Additionally, PIF4 forms a regulatory module with *RBOHD* and *FLS2* to coordinate ROS signalling in response to drought and salt stress (Liu et al. 2022). However, the specific mechanisms by which *StPIF4* regulates ROS under high‐temperature stress remain unclear. This study preliminarily confirmed that StPIF4 enhances ROS scavenging capacity in potato under high‐temperature stress, yet its downstream pathways have not been fully elucidated. Previous studies have reported that *PIF4* participates in regulating auxin‐related gene expression during thermomorphogenesis (Li et al. 2021), and auxin can modulate ROS homeostasis (Tognetti et al. 2012). This provides a key clue for investigating whether *StPIF4* regulates ROS balance by modulating auxin biosynthesis, transport, or signalling, which awaits further genetic and molecular validation.

This study established a high‐resolution single‐nucleus transcriptomic atlas of potato leaf heat stress responses, providing the first cellular‐resolution characterisation of spatiotemporal regulatory mechanisms coordinating leaf development and thermotolerance. Through systematic analysis of heat‐responsive gene dynamics, pseudo‐time trajectory reconstruction, and core regulatory networks, we elucidated early molecular response signatures in heat‐stressed potato leaves. This atlas deciphers the molecular regulatory framework governing crop cell differentiation under heat stress through a cellular heterogeneity lens, establishing a foundation for thermotolerant germplasm innovation and precision breeding.

## 4 Experimental Procedures

### 4.1 Plant Material and Heat Stress Treatment

The doubled haploid potato line ‘DM 1‐3516 R44’ was used as the research material for snRNA‐seq and cultivated in an artificial climate chamber. The plants were cultivated under constant day/night temperatures of 22°C, a photoperiod of 16 h light/8 h darkness, and 55% relative humidity for 14 days before being subjected to heat stress. For the heat stress, plants were exposed to 35°C during the day and 28°C at night, with relative humidity maintained at 55%. Following 24 h of continuous treatment, leaf samples were harvested and flash‐frozen in liquid nitrogen for subsequent snRNA‐seq analysis.

Potato cultivars ‘Atlantic’ (AT) and ‘E potato 3’ (E3) were used as the recipient materials for generating *StPIF4* overexpression and RNAi transgenic lines, respectively. Additionally, E3 was used for generating the *proStPIF4*::GUS transgenic reporter line. Fifteen‐day‐old in vitro wild‐type and transgenic plants were transplanted into potting mix and grown in a controlled climate chamber under the following conditions: 22°C day/night, 16 h light/8 h dark photoperiod. After 30 days of growth, plants were subjected to heat stress (42°C day/night), while control plants remained at 22°C. Phenotypes were observed and photographed following 48 h of heat stress.

### 4.2 Potato Leaf Nuclei Isolation

Nuclei isolation was performed following a modified protocol based on the 10× Genomics ‘Nuclei Isolation for Single Cell RNA Sequencing’ guidelines (CG000365, Rev. C). Fresh potato leaves were added to 1 mL ice‐cold lysis buffer and finely minced into ~1 mm fragments. An additional 2 mL of pre‐chilled lysis buffer was added, and the mixture was transferred into a Kimble Dounce Homogenizer (885300–0007). Samples were gently homogenised with 5 slow strokes using the loose (A) pestle. The homogenate was incubated on ice for 5 min to ensure complete cell lysis. Subsequently, 4 mL of ice‐cold 2% BSA was added to stop the reaction, followed by gentle mixing using a wide‐bore pipette. The suspension was filtered through a 30 μm cell strainer and the flow‐through was collected. Filtrates were centrifuged at 300 *g* for 5 min at 4°C, and the supernatant was discarded. The pellet was washed once with 5 mL of 2% BSA, followed by centrifugation at 300 *g* for 5 min at 4°C. The final nuclei pellet was resuspended in 500 μL nuclei resuspension buffer (1× PBS, 2% BSA, 0.1% RNase inhibitor). To remove debris and organelle contamination, nuclei were stained with Propidium Iodide (PI, 1 μg/mL) and sorted on a BD FACSAria II using a 70 μm nozzle, gating for PI‐positive, debris‐free nuclei. Sorted nuclei were collected into tubes pre‐coated with 2% BSA and counted using a haemocytometer/automated counter. Preparations yielding > 90% intact, debris‐free nuclei were used for snRNA‐seq.

### 4.3 SnRNA‐Seq Library Construction and Sequencing

Single‐nucleus RNA‐seq (snRNA‐seq) libraries were prepared using the Chromium Single Cell 3′ Reagent Kits v3.1 (10× Genomics, PN‐1000268) according to the manufacturer's instructions (CG000204). Approximately 8000 nuclei were loaded per channel of a Chromium Next GEM Chip G to generate Gel Bead‐in‐Emulsions (GEMs). Reverse transcription inside GEMs was performed using the v3.1 RT master mix, producing barcoded cDNA from individual nuclei. After GEM cleanup, cDNA amplification was performed for 12 cycles, following the standard cycling conditions specified by the manufacturer. Amplified cDNA was quality‐checked using an Agilent 2100 Bioanalyzer (expected distribution: 400–2000 bp). Samples with no signs of genomic contamination or degradation were used for library preparation. Sequencing libraries were constructed using the Chromium Single Cell 3′ Library Kit v3.1, following fragmentation, end‐repair, A‐tailing, adaptor ligation, and sample index PCR (10 cycles). Final libraries were assessed using the Bioanalyzer High Sensitivity DNA Kit to confirm appropriate size distribution (~350 bp). Libraries were sequenced at LC‐Bio Technology (Hangzhou, China) on an Illumina NovaSeq 6000 platform using paired‐end 150 bp (PE150) reads with the following read configuration per 10× recommendations: Read 1: 28 bp (cell barcode + UMI); Read 2: 91 bp (cDNA insert). A minimum sequencing depth of ≥ 20 000 reads per nucleus was ensured.

### 4.4 Raw Data Processing and Differential Gene Expression Analysis

Raw base call (BCL) files from Illumina sequencing were demultiplexed and converted to FASTQ format using bcl2fastq software (v5.0.1). The snRNA‐seq data were aligned to the potato reference genome using CellRanger software (v7.2.0, 10× Genomics), and gene expression counts per nucleus (based on 3′ end transcripts) were generated in the sequenced samples (recommended sequencing depth of > 20 000 reads per cell, support.10xgenomics.com). Process barcodes associated with cell identifiers and unique molecular identifiers (UMIs) using the Seurat R package (v4.1.0) in R (v3.6.0). Cells with mitochondrial UMI counts > 25% or < 500 genes were identified as low‐quality and removed. Following doublet removal using DoubletFinder (v2.0), the gene expression matrix was normalised using a standardisation function, and the top 2000 features with high inter‐cell variability were calculated.

Dimensionality reduction was performed using PCA followed by UMAP. Nuclei were visualised in the resulting low‐dimensional space, and cell clusters were identified. Seurat was used to identify DEGs between cell clusters. DEGs were defined using the following thresholds: |log2 fold change (FC)| > 0.35, minimum expression fraction (proportion of nuclei expressing the gene within a cluster) ≥ 0.1, and adjusted *p*‐value < 0.05. Additionally, DEGs between control and heat stress samples within each cell cluster were identified using thresholds of |log2FC| > 0.5 and adjusted *p*‐value < 0.05. The ‘FindAlMarkers’ function in Seurat identified DEGs specific to each cluster (only.pos = FALSE, min.pct = 0.1, thresh. use = 0.1) and performed hypergeometric test GO and KEGG pathway enrichment analysis.

### 4.5 Pseudo‐Time Trajectory Analysis

The pseudo‐time trajectory analysis included trajectory construction, differential gene expression analysis, and branch point identification. Cell pseudo‐time trajectories were constructed using the R package Monocle (v2.4) with default parameters. First, pseudo‐times were sorted using the ‘reduceDimension’ function, setting the maximum principal component count to 2 and applying the DDRTree dimension reduction method. Next, the ‘differentialGeneTest’ function (fullModelFormulaStr = ~Pseudotime) was used to identify genes exhibiting significant changes across pseudo‐time, selected from the top 50 marker genes within each cluster. Finally, the ‘plot_pseudotime_heatmap’ function was used to visualise these genes. Developmental trajectories branch at specific pseudo‐time points, representing fate choice points.

### 4.6 Bulk RNA‐Seq Library Construction and Sequencing

Total RNA was extracted from using TRIzol reagent according to the manufacturer's instructions. Three biological replicates were prepared per sample. The ligation reaction products were purified using AMPure XP Beads (1.0× ratio). The purified products were amplified by polymerase chain reaction (PCR). The resulting cDNA libraries were sequenced on an Illumina NovaSeq6000 platform by Gene Denovo Biotechnology Co. (Guangzhou, China). Clean reads were aligned to the reference genome using HISAT2 (v.2.2.4) (Kim et al. 2015). Transcript expression levels were quantified expression using StringTie (v1.3.1) and RSEM (v1.3.3) (Li and Dewey 2011; Pertea et al. 2015).

### 4.7 RNA Fluorescence In Situ Hybridization

Potato leaves from control plants were harvested and immediately fixed in FAA solution (50% ethanol, 5% acetic acid, and 3.7% formaldehyde) for > 12 h. Antisense RNA probes targeting specific genes were synthesised using complementary DNA (cDNA) as template, following the DIG High Prime DNA Labeling and Detection Kit protocol (Roche). Primers used for probe synthesis are listed in Table S21. RNA fluorescence in situ hybridization was performed as previously described (Denyer et al. 2019). Images were acquired using a LeicaMZ10Fmicroscope (Leica Co., Baden‐Württemberg, Germany).

### 4.8 Potato Genetic Transformation

To construct the overexpression vector, the coding sequence (CDS) of *StPIF4* was cloned into the pCAMBIA1300 vector, yielding the recombinant *35S*::StPIF4‐GFP construct. For the RNAi vector, a specific fragment of the *StPIF4* CDS was inserted into the pHELLSGATE‐8 vector. The reporter construct *proStPIF4*::GUS was generated by cloning a 2000‐bp promoter region upstream of the ATG start codon of *StPIF4* into the pBI101‐GUS vector. Transgenic potato plants were subsequently obtained via Agrobacterium‐mediated transformation of potato tuber discs, following a previously described method (Gangadhar et al. 2016). Positive transformants were initially screened by PCR, and the transformation efficiency was further validated by quantitative real‐time PCR (qRT‐PCR).

### 4.9 DNA Extraction and qRT‐PCR

Genomic DNA was extracted using the CTAB method (Beijing Coolaber Science & Technology Co. Ltd., Cat# SL 2071) according to the manufacturer's instructions. qRT‐PCR was performed on a Bio‐Rad CFX96 system using TB Green Premix Ex Taq II FAST qPCR Kit (Takara, Cat# CN830S). The *StEF1α* gene was used as an internal reference for all qRT‐PCR experiments, and relative gene expression levels were calculated using the 2^−ΔΔCT^ method (Livak and Schmittgen 2001). All primers used are listed in Table S21.

### 4.10 GUS Staining

The GUS staining solution was prepared as follows: 50 mM PBS containing 0.2% Triton X‐100, 5 mM potassium ferrocyanide, 5 mM potassium ferricyanide, and 2 mM X‐Gluc (Yeasen Biotechnology (Shanghai) Co. Ltd., Cat# 10904ES03). Leaf samples from potato plants subjected to 42°C for 48 h were immersed in the GUS staining solution and placed in a vacuum desiccator for 30 min. Subsequently, the samples were incubated at 37°C in the dark for 12 h. After incubation, the staining solution was discarded, and the leaves were decolorized with 95% ethanol. Staining results were observed and photographed. Three biological replicates were performed for each experiment.

### 4.11 Measurement of Physiological Indices and Relative Electrolyte Leakage

After 30 days of growth, wild‐type and *StPIF4* transgenic potato were exposed to either control (22°C) or heat stress (42°C) for 48 h. Leaf samples were then collected for physiological assays. Superoxide dismutase (SOD), peroxidase (POD), catalase (CAT), and malondialdehyde (MDA) levels were measured using commercial assay kits (Grisp Biological Science and Technology Co. Ltd., Suzhou; Cat# G0101W for SOD, G0107W for POD, G0105W for CAT, and G0109W for MDA) according to the manufacturer's instructions. Three biological replicates were performed for each assay.

For relative electrolyte leakage assessment, fully expanded leaves from WT, *StPIF4*‐RNAi, and *StPIF4*‐OE lines were collected after 48 h under CK or 42°C heat stress. Twenty leaf discs were obtained using a punch tool, placed in a tube containing 10 mL of deionised water, and incubated at room temperature for 4 h. The initial conductivity (R1) was measured, after which the samples were boiled for 40 min to disrupt the tissues completely. After cooling to room temperature, the final conductivity (R2) was measured. Relative electrolyte leakage was calculated as (R1/R2) × 100%. Three biological replicates were included.

### 4.12 DAB, NBT, and Trypan Blue Staining

DAB (0.1%), NBT (0.05%), and Trypan blue (0.05%) staining solutions were prepared using commercial reagents (Beijing Coolaber Technology Co. Ltd.; Cat# CD4181, CN7731, and CT11481, respectively). Leaf samples of consistent size and position were collected from potato plants subjected to either control (22°C) or 48 h HT stress (42°C) and immersed in the respective staining solutions for 12 h. After complete decolorization with ethanol, the stained leaves were photographed and documented. Three biological replicates were performed for each treatment.

### 4.13 Dual‐Luciferase Assay (Luc)

The encoding sequence (CDS) of *StERF5*, *StRL1*, *StKUA1*, and *StWRKY40* was cloned into the pCAMBIA1300 vector under the control of the CaMV 35S promoter. The promoter sequences (2000 bp) of *StGGCT1.2*, *StSAP5*, *StFT*, *StRD21B*, and *StRALF* were cloned into the reporter vector pGreenII 0800‐LUC (containing a 35S::REN cassette for normalisation). Following transient expression in tobacco leaves via *Agrobacterium*‐mediated transformation, dual‐luciferase activity was measured using the Dual‐Luciferase Reporter Assay System (Promega, Catalogue #E1910) according to the manufacturer's instructions. Primers used for cloning are listed in Table S22. The experiment was performed with three independent biological replicates.

### 4.14 Electrophoretic Mobility Shift Assay (EMSA)

The StPIF4 coding sequence was cloned into the pET‐32a‐GST vector, and the recombinant plasmid was expressed in *Escherichia coli* Rosetta (DE3). The GST‐StPIF4 protein was induced by IPTG for 16 h at 18°C and purified using a Ni‐NTA agarose kit (Qiagen). *StHSFA2* 50 bp oligonucleotide probes were synthesised by Sangon Biotech (Shanghai, China) and labelled with biotin at the 3′ end. Mutant probes were generated and labelled by replacing ‘AAAAA’ with ‘g’. EMSA was performed using the Light‐Shift Chemiluminescent EMSA Kit (GS009; Beyotime Biotech, Shanghai, China) according to the manufacturer's instructions.

## Author Contributions

D.L., H.J. and S.W. designed the experiments. S.W., K.W., W.L., R.L. and Z.L. completed the experiments. S.W. carried out the data analysis. S.W., K.W. and W.L. wrote the manuscript. H.J., X.C. and Y.L. gave advice and edited the manuscript. All authors read and approved the final manuscript.

## Conflicts of Interest

The authors declare no conflicts of interest.

## Supporting information


**Figure S1:** Visualisation of potato leaf snRNA‐seq analysis using t‐SNE.
**Figure S2:** Cellular repopulation analysis by PCs and PLCs.
**Figure S3:** Identification of core TFs for pseudo‐time trajectory analysis.
**Figure S4:** Transcriptional co‐expression network of potato leaves in response to heat stress at single‐cell resolution.
**Figure S5:** Identification of transformation efficiency in transgenic potato.
**Figure S6:** StPIF4 directly binds to the *StHSFA2* promoter and enhances transcriptional activity.

**Table S1:** Statistics of preliminary mapping results of snRNA‐seq data.
**Table S2:** Statistical computation of cell clusters.
**Table S3:** The reported marker genes for cell clusters.
**Table S4:** The GO enrichment results of marker genes for cell clusters.
**Table S5:** Novel marker genes for cell clusters in potato.
**Table S6:** Top 5 novel marker genes in different cell clusters.
**Table S7:** Statistical table of the distribution information of subpopulations in cell trajectories.
**Table S8:** Pseudo‐time trajectory branch Node I & II GO enrichment analysis results.
**Table S9:** Expression matrix of up‐regulated DEGs under heat stress in PCs and PLCs.
**Table S10:** GO enrichment analysis results of up‐regulated DEGs under high temperature stress in PCs and PLCs.
**Table S11:** The core TFs in each cell cluster.
**Table S12:** The DEGs of each cell cluster between CK and heat‐stress samples.
**Table S13:** The core TFs of each cell cluster between CK and heat‐stress samples.
**Table S14:** The core TFs of EC clusters between CK and heat‐stress samples.
**Table S15:** Trajectory analysis identified the DEGs along with the timeline.
**Table S16:** Identification of DEGs in all cell differentiation states.
**Table S17:** Identification of DEGs in different cell differentiation branches.
**Table S18:** The differentially expressed TFs in bulk RNA‐seq.
**Table S19:** The hub TFs in WGCNA.
**Table S20:** Co‐expression network of candidate core TFs within the brown module.
**Table S21:** The primer sequences used for probe synthesis probe of in situ hybridization.
**Table S22:** The primers used in this study.

## Data Availability

The raw sequence datasets from this research have been submitted to the NGDC GSA database under the project numbers: PRJCA042819 and PRJCA042859. The data are publicly accessible at the following URL: https://ngdc.cncb.ac.cn/gsa.
