# Supplementary material for: Transcription Profiling of Potato Leaves in Response to Heat Stress at Single‐Cell Resolution
Source: Plant Biotechnol J. 2026 Jan 13;24(5):2970–87. doi: 10.1111/pbi.70546 (PMC13110171; doi:10.1111/pbi.70546)
Supplement: Supplementary file 1 — Figure S1: Visualisation of potato leaf snRNA‐seq analysis using t‐SNE. Figure S2: Cellular repopulation analysis by PCs and PLCs. Figure S3: Identification of core TFs for pseudo‐time trajectory analysis. Figure S4: Transcriptional co‐expression network of potato leaves in response to heat stress at single‐cell resolution. Figure S5: Identification of transformation efficiency in transgenic potato. Figure S6: StPIF4 directly binds to the StHSFA2 promoter and enhances transcriptional activity. [file PBI-24-2970-s002.docx]

**
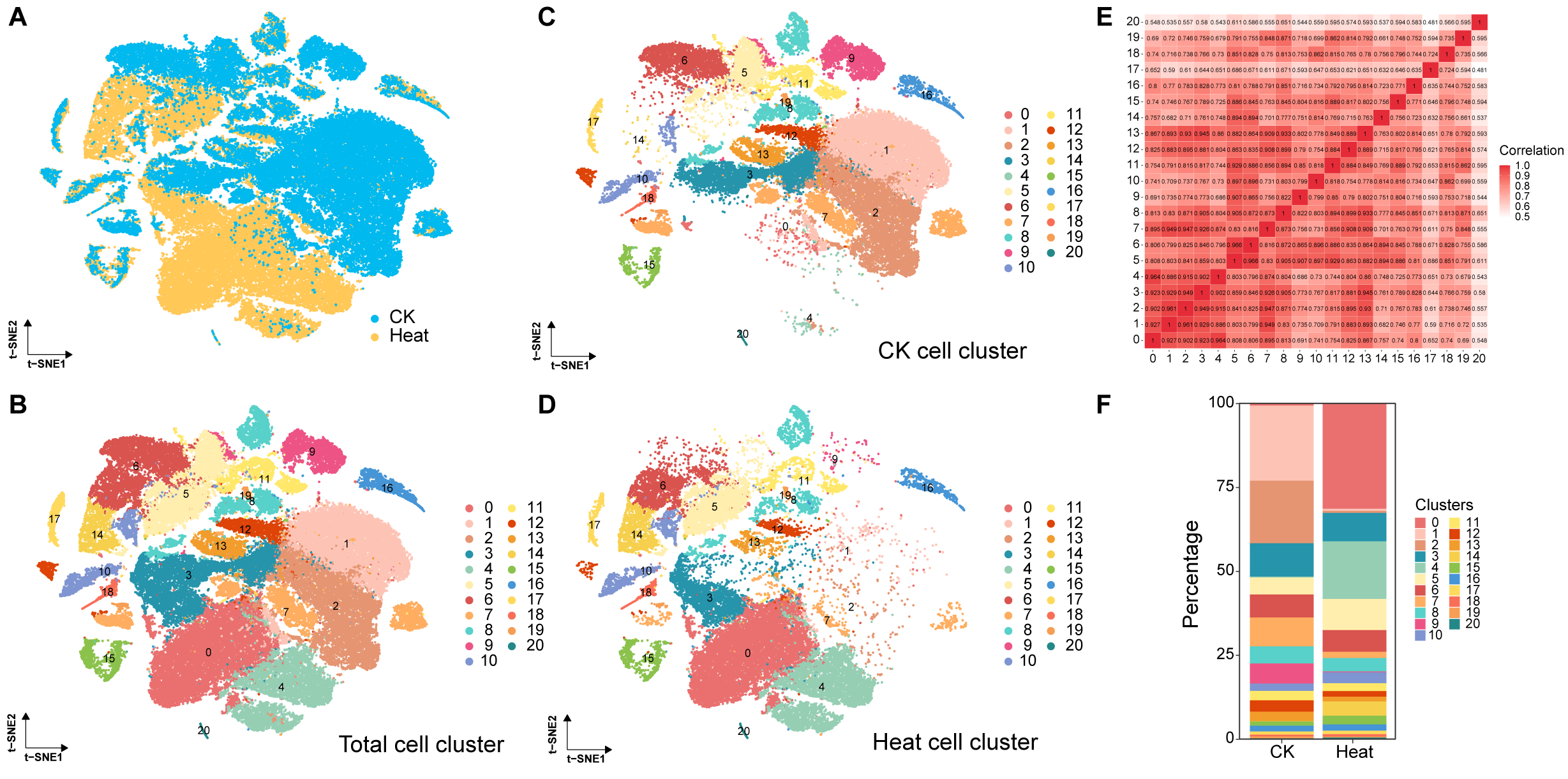
**

**Figure S1.** **Visualization of potato leaf snRNA-seq analysis using t-SNE.** (A) T-SNE plot illustrated leaf cell clusters of CK and heat-stressed seedlings. Each dot represented a cell. The cells from CK and heat-stressed seedlings were represented in blue and yellow color, respectively. (B) 21 colors represented 21 distinct cell clusters identified snRNA-seq. (C, D) Leaf cell clusters of CK and heat-stressed samples, respectively. (E) Pearson correlation analysis between clusters. The darker the color, the stronger the correlation. (F) Histogram of cell count statistics for each cluster in CK and heat-stressed. The number of cells in different. 21 colors represented 21 distinct cell clusters identified snRNA-seq. The t-SNE visualization results were provided here for comparative reference.

**
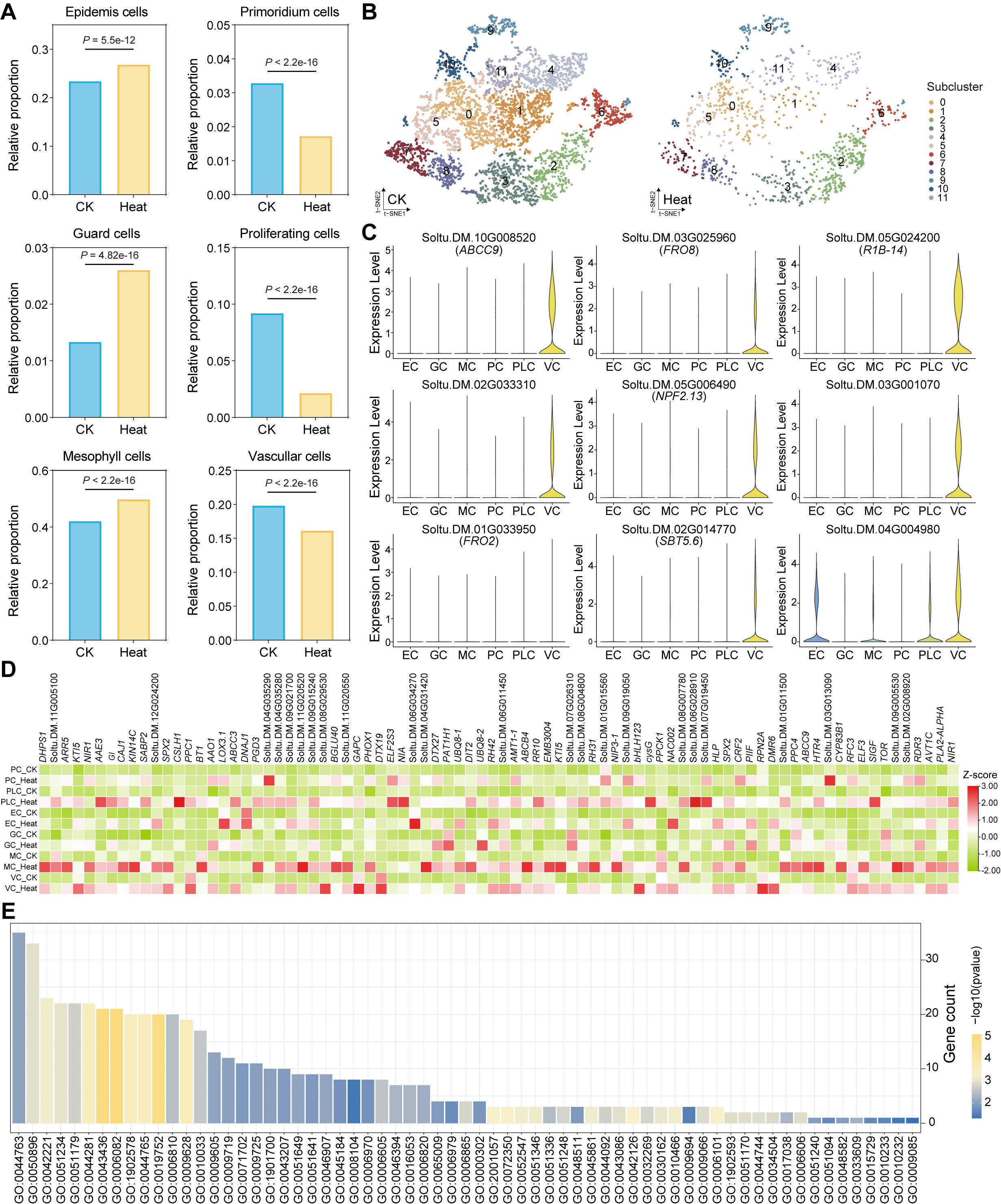
**

**Figure S2.** **Cellular repopulation analysis by PCs and PLCs.** (A) The proportionality test of six cell types under CK and heat stress. (B) PCs and PLCs for t-SNE visualization. Left, CK samples; right, heat samples. (C) Subcluster 10 overlapping marker genes expression minutiae in different cell types. (D) Heatmap of cell type expression of 82 subcluster-specific marker genes differentially up-regulated at heat stress. (E) Go enrichment analysis of 82 subcluster-specific marker genes that were differentially up-regulated at heat stress. *P*-value <0.05.


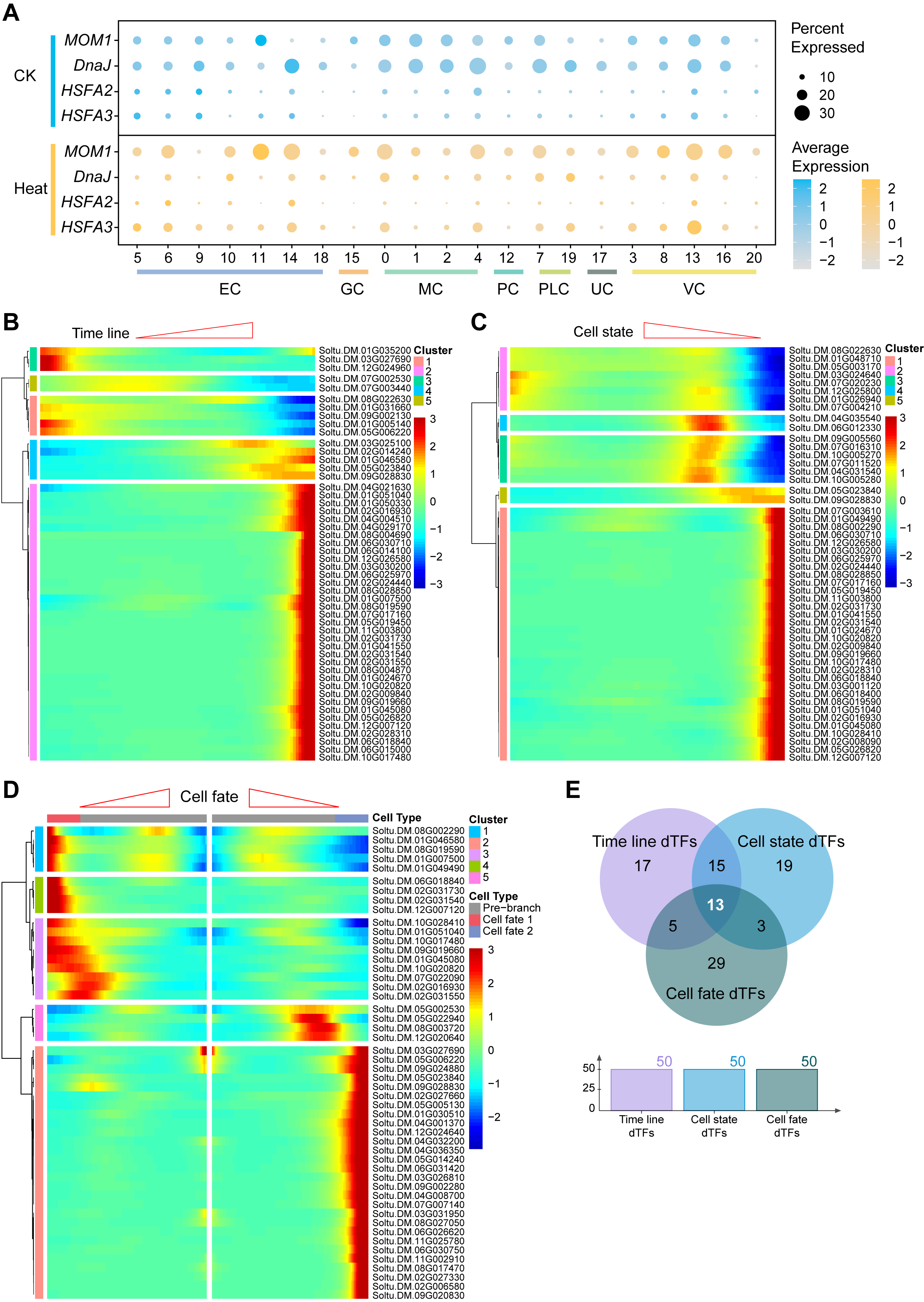


**Figure S3. Identification of core TFs for pseudo-time trajectory analysis.** (A) Expression patterns of heat-stress relative genes for ECs in CK and heat-stress samples, respectively. Bubble plots and color gradients illustrates the expression pattern and distribution of these representative genes. Standardized average expression levels using the Z-Score method. (B) Clustering and expression patterns of top 50 TFs of cell differentiation along the pseudo-time progression. (C) Expression of top 50 TFs involved in cell differentiation states. (D) Expression of top 50 TFs involved in cell differentiation states. (E) The Venn diagram indicated core and specific TFs from cell differentiation, cell state differentiation and cell fate.


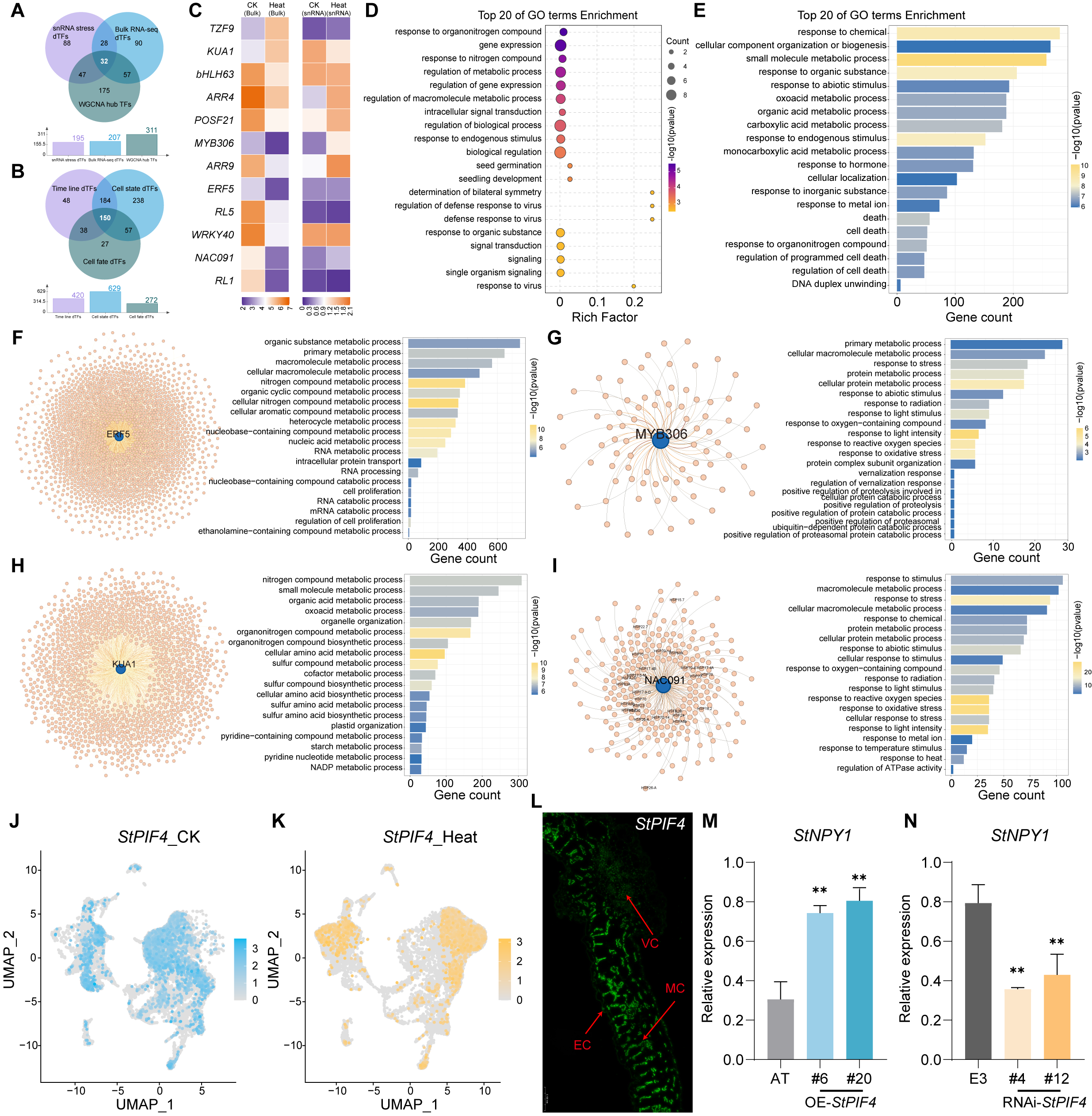


**Figure S4. Transcriptional co-expression network of potato leaves in response to heat stress at single-cell resolution.** (A) The Venn diagram indicated core and specific TFs from bulk RNA-seq and snRNA-seq. (B) The Venn diagram indicated core and specific TFs from cell differentiation, cell state differentiation and cell fate. (C) Expression heatmaps for the 12 core TFs are in bulk RNA-seq and snRNA-seq, respectively. Standardized average expression levels using the Z-Score method. (D) Top 20 GO terms in 12 core TFs. (E) Top 20 go enrichment results of candidate target genes for WGCNA brown module core TFs. (F–I) Co-expression network of core TFs and top 20 GO enrichment results of candidate target genes. Blue dots: candidate core TFs within the brown module; Orange dots: corresponding co-expressed genes of the candidate core TFs; Dot size: degree; Edges: linking candidate core TFs and their co-expressed genes at either end, forming co-expression gene pairs. As the weight increases, edges become thicker and darker in color. (J) UMAP expression map of *StPIF4* under CK conditions. (K) UMAP expression map of *StPIF4* under heat conditions. (L) RNA *in situ* hybridization assays for *StPIF4* in the potato leaf. In the *in situ* hybridization antisense images, the identified cell types were indicated by red arrowheads. Bars, 100 μm. (M, N) qRT-PCR identification of *StNPY1* in *StPIF4*-OE lines (M) and *StPIF4*-RNAi lines (N). Data were means ± SEM (n = 3). Statistical method using one-way ANOVA, *, *P*-value <0.05; **, *P*-value <0.01.


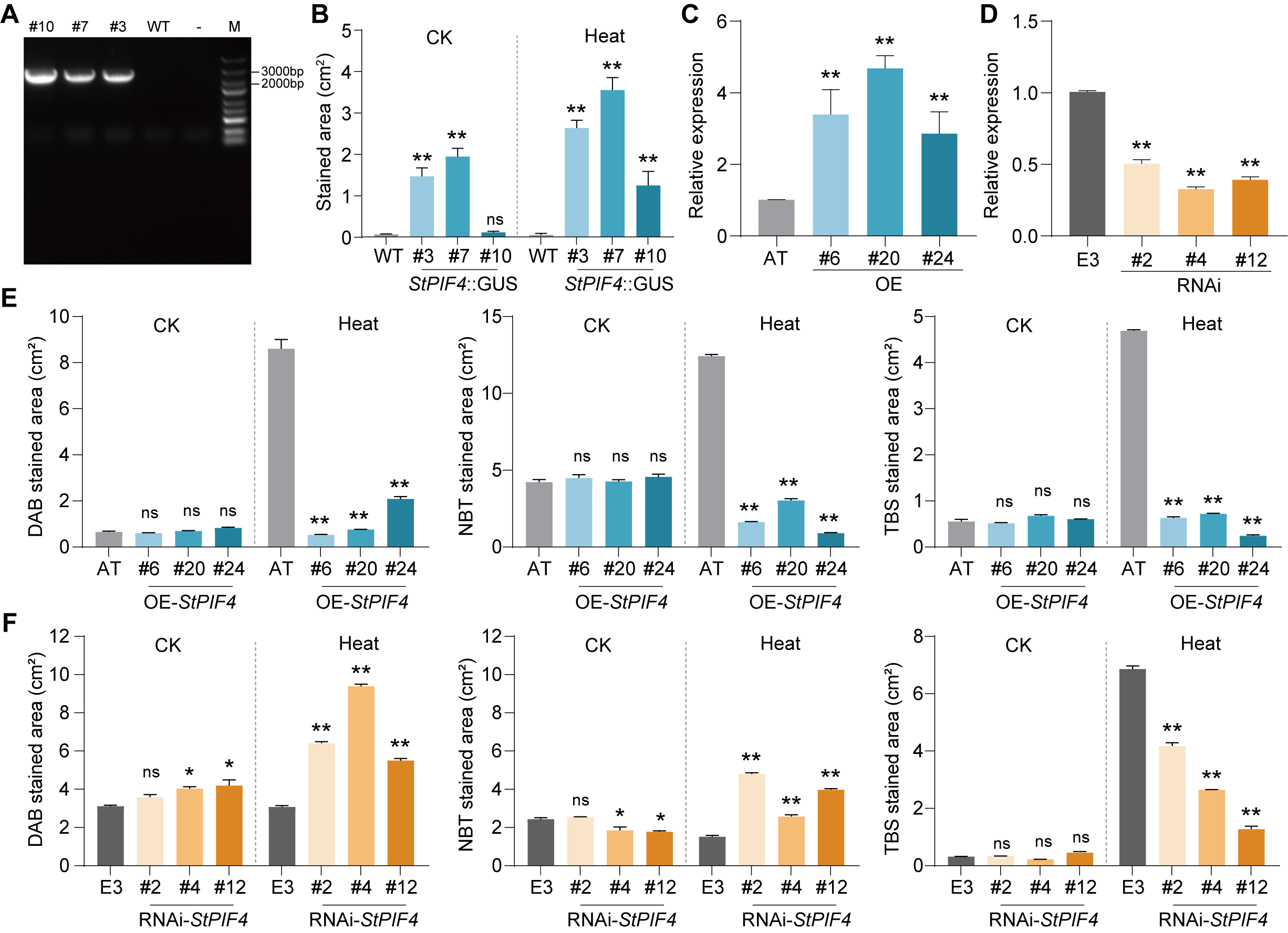


**Figure S5. Identification of transformation efficiency in transgenic potato.** (A) Identification of *StPIF4* promoter transgenic by PCR. (B) Statistics of GUS-stained areas in potato leaves expressing *StPIF4* under CK and heat stress conditions, n = 2. (C) qRT-PCR identification of overexpression of *StPIF4* transgenic potato. (D) qRT-PCR identification of RNAi of *StPIF4* transgenic potato. Data were means ± SEM of three biological replicates. Statistical method using one-way ANOVA, *, *P*-value <0.05; **, *P*-value <0.01. (E-F) Statistics of DAB, NBT, and trypan blue staining areas in potato leaves of *StPIF4* OE lines (E)and RNAi lines (F)under CK and heat stress conditions. Data were means ± SEM (n = 2). DAB, diaminobenzidine; NBT, nitrotetrazolium blue chloride; TBS, trypan blue. Statistical method using one-way ANOVA, *, *P*-value <0.05; **, *P*-value <0.01; ns, non-significant.


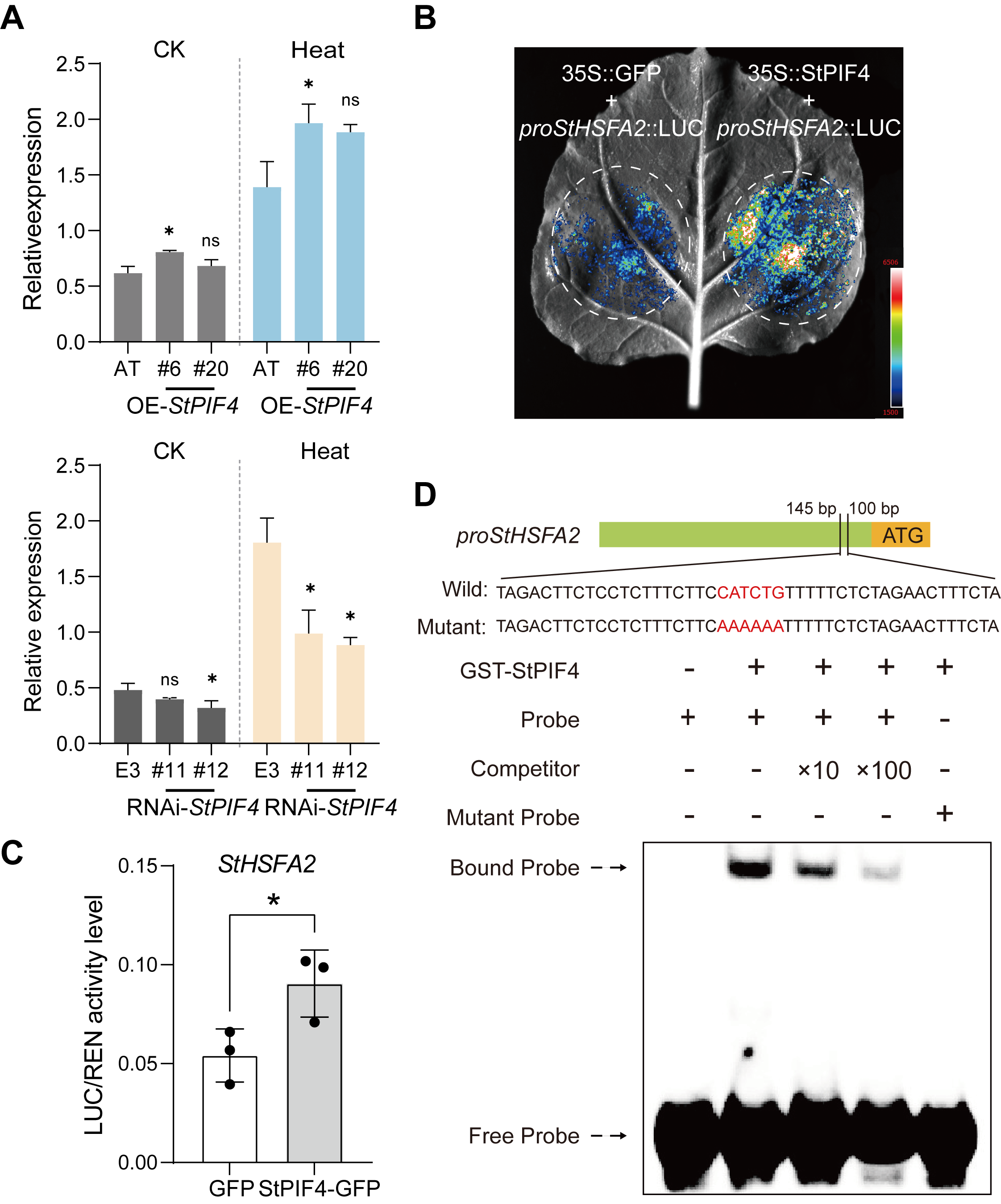


**Figure S6. StPIF4 directly binds to the *StHSFA2* promoter and enhances transcriptional activity.** (A) qRT-PCR identification of *StHSFA2* in *StPIF4*-OE lines (top) and *StPIF4*-RNAi lines (bottom). Data were means ± SEM (n = 3). Statistical method using one-way ANOVA, *, *P*-value <0.05; **, *P*-value <0.01; ns, non-significant. (B) Dual-luciferase assay of StPIF4 and *StHSFA2*. (C) Statistical analysis of *StHSFA2* luciferase activity. Data are presented as mean ± SEM (n = 3). Statistical method using one-way ANOVA. *, *P*-value <0.05. (D) EMSA probe of *StHSFA2* design and EMSA assay of StPIF4 with promoter of *StHSFA2*.
